# Extreme Genetic Fragility of the HIV-1 Capsid

**DOI:** 10.1371/journal.ppat.1003461

**Published:** 2013-06-20

**Authors:** Suzannah J. Rihn, Sam J. Wilson, Nick J. Loman, Mudathir Alim, Saskia E. Bakker, David Bhella, Robert J. Gifford, Frazer J. Rixon, Paul D. Bieniasz

**Affiliations:** 1 Aaron Diamond AIDS Research Center, The Rockefeller University, New York, New York, United States of America; 2 Laboratory of Retrovirology, The Rockefeller University, New York, New York, United States of America; 3 Howard Hughes Medical Institute, Aaron Diamond AIDS Research Center, New York, New York, United States of America; 4 MRC Centre for Virus Research, Institute of Infection, Immunity and Inflammation, College of Medical, Veterinary and Life Sciences, University of Glasgow, Glasgow, United Kingdom; 5 Centre for Systems Biology, University of Birmingham, Birmingham, United Kingdom; University of Massachusetts Medical School, United States of America

## Abstract

Genetic robustness, or fragility, is defined as the ability, or lack thereof, of a biological entity to maintain function in the face of mutations. Viruses that replicate via RNA intermediates exhibit high mutation rates, and robustness should be particularly advantageous to them. The capsid (CA) domain of the HIV-1 Gag protein is under strong pressure to conserve functional roles in viral assembly, maturation, uncoating, and nuclear import. However, CA is also under strong immunological pressure to diversify. Therefore, it would be particularly advantageous for CA to evolve genetic robustness. To measure the genetic robustness of HIV-1 CA, we generated a library of single amino acid substitution mutants, encompassing almost half the residues in CA. Strikingly, we found HIV-1 CA to be the most genetically fragile protein that has been analyzed using such an approach, with 70% of mutations yielding replication-defective viruses. Although CA participates in several steps in HIV-1 replication, analysis of conditionally (temperature sensitive) and constitutively non-viable mutants revealed that the biological basis for its genetic fragility was primarily the need to coordinate the accurate and efficient assembly of mature virions. All mutations that exist in naturally occurring HIV-1 subtype B populations at a frequency >3%, and were also present in the mutant library, had fitness levels that were >40% of WT. However, a substantial fraction of mutations with high fitness did not occur in natural populations, suggesting another form of selection pressure limiting variation *in vivo*. Additionally, known protective CTL epitopes occurred preferentially in domains of the HIV-1 CA that were even more genetically fragile than HIV-1 CA as a whole. The extreme genetic fragility of HIV-1 CA may be one reason why cell-mediated immune responses to Gag correlate with better prognosis in HIV-1 infection, and suggests that CA is a good target for therapy and vaccination strategies.

## Author Summary

The HIV-1 capsid protein (CA) is absolutely essential for viral replication and there is, therefore, intense evolutionary pressure for HIV-1 CA to conserve its functions. However, HIV-1 CA is also a key target of the host immune response, which should provide evolutionary pressure to diversify CA sequence. Genetic robustness, or fragility, is defined as the ability, or lack thereof, of a biological entity to preserve function in the face of sequence changes. Thus, it should be advantageous to HIV-1 CA to evolve genetic robustness. Here, we present the results of extensive, random mutagenesis of single amino acids in CA that reveal an extreme genetic fragility. Although CA participates in several steps in HIV-1 replication, the biological basis for its genetic fragility was primarily the need to participate in the efficient and proper assembly of mature virion particles. The extreme genetic fragility of HIV-1 CA may be one reason why immune responses to it correlate with better prognosis in HIV-1 infection, and suggests that CA is a good target for therapy and vaccination strategies.

## Introduction

Genetic robustness is defined as the ability of a biological entity (e.g. a protein or organism) to maintain function in the face of mutations [1], [2]. More robust proteins or organisms tolerate higher mutation rates while less robust (more ‘brittle’ or ‘fragile’) proteins or organisms are intolerant of mutation and are more likely to lose function or be driven to extinction by high mutation rates. Viruses that replicate via RNA intermediates using non-proofreading polymerases exhibit high mutation rates, suggesting that robustness should be particularly advantageous to them [3]. Indeed, under some conditions, viral populations that exhibit high robustness at the expense of high fitness might be favored over those that have high fitness but low robustness [4]–[6]. In other words, robustness/fragility might be a more potent selective force than fitness under some circumstances.

While the robustness or fragility of RNA viruses has been investigated in several studies [4]–[9], most reports characterize robustness by treating an entire viral genome as a single biological entity. However, viruses encode a variety of proteins that execute a range of functions to enable replication, and the genetic robustness of individual proteins is expected to vary within a given virus. Even within a single protein, individual domains may exhibit variation in robustness/fragility. Proteins that perform complex or multiple functions that are highly dependent on accurate structure (e.g. enzymes), should tend to be more genetically fragile, i.e less tolerant of mutation, and exhibit greater sequence conservation than those that do not (e.g. proteins that simply provide peptide binding sites to recruit other proteins).

However, in their natural setting, i.e. a susceptible host, animal viruses often replicate in a hostile and changing environment, shaped partly by adaptive immune responses. Such immune responses can take the form of antibodies that target proteins displayed on the surface of the virion, or cytotoxic lymphocytes that can target epitopes in nearly any viral protein. Hence, at least some viral proteins, even those that should exhibit genetic fragility, are placed under strong evolutionary pressure to diversify in sequence, because they are targeted by adaptive immune responses. Under such conditions, otherwise fragile proteins might be expected to evolve higher robustness. However, the potential trade-off between robustness and fitness might impinge on each property, and it is unclear whether simple fitness, or the acquisition of robustness, would constitute the dominant selective force on a given viral protein in a natural setting.

One such protein in which the competing needs to preserve function yet diversify sequence might conflict in a particularly acute manner, is human immunodeficiency virus type I (HIV-1) capsid protein (CA). As a critically important viral protein, it is placed under strong selective pressure to maintain its structure and perform several functions. Conversely, as a highly expressed, immunologically visible protein, CA also experiences a competing pressure to diversify its sequence. CA exists both as a domain within the Gag precursor polyprotein during particle assembly, and as an autonomous protein in the mature virion. Both Gag and CA are multifunctional proteins [10]–[13]. Specifically, during virion morphogenesis, Gag molecules assemble at the plasma membrane and drive the formation of roughly spherical immature virions, containing radially arrayed Gag molecules, that bud through the plasma membrane [14], [15]. However, during and after viral budding, the viral protease (PR) is activated and catalyses the cleavage of Gag at five positions, causing profound morphological transformations [13]. In particular, the liberated CA protein forms a conical capsid that encapsulates the nucleocapsid-genomic RNA complex [16], [17].

Like all retroviral CA proteins, a single HIV-1 CA molecule is composed of two domains: the N-terminal domain (NTD) comprised of 146 amino acids, and C-terminal domain (CTD) comprised of 85 amino acids. The NTD structure consists of an N-terminal β hairpin and 7 succeeding α helices, while the CTD has 4 α helices, and a C-terminal unstructured region of 11 residues [18]–[20]. The NTD and CTD are joined by an interdomain linker region (residues 146–150). Other noteworthy features of CA include the major homology region (MHR residues 153–172) of the CTD, which is a highly conserved 20 amino-acid region found in all retroviruses [21] and the likely binding site of ABCE1 [22], and a loop (NTD residues 85–93) which binds the cellular protein cylophilin A [23]. The mature HIV-1 capsid consists of ∼1100 CA monomers assembled into a hexameric lattice with 12 pentameric declinations [16], [24], that are distributed in such a way that the viral capsid takes the form of a fullerene cone. Within the lattice, the NTD is primarily responsible for intra-hexamer contacts, while the CTDs form dimers that link adjacent hexamers [25], [26]. However, interactions between the NTD and CTD also contribute to proper capsid formation [26]–[29].

In addition to its major structural role, CA is a key determinant of several other biological properties of HIV-1. For example, HIV-1 capsid is the key determinant that enables HIV-1 to infect non-dividing cells [30], [31]. Related work has identified genetic or physical interactions with host proteins karyopherin β transportin-3 (TNPO3), nucleoporin 153 (NUP 153), nucleoporin 358 (NUP358)/RanBP2 and cleavage and polyadenylation factor 6 (CPSF6) [32]–[36]. Moreover, interactions between HIV-1 capsid and cyclophilin A influence nuclear import and subsequent integration site selectivity, as well as replication efficiency, in a cell type dependent manner [37]–[40].

CA is a key target of intrinsic, innate and adaptive immune defenses. Specifically, CA is targeted by TRIM5α [41], and may be detected by undefined sensors in dendritic cells [42]. HIV-1 CA also adapts under immune selection *in vivo* [43]–[46]. In particular, host CD8+ cytotoxic T lymphocyte (CTL) responses to HIV-1 infection are critical determinants of viral control [47], [48], and there is an association between Gag- and capsid-specific CD8+ T-cell responses and *in vivo* viral burden[49]. The emergence of viral ‘escape’ mutations in CA can result in higher viremia [50], [51]. However, escape mutations often incur a significant fitness cost, which may be subsequently compensated for by secondary mutations in CA, or drive reversion when immune pressure is lifted [45], [51], [52].

Previous studies in which CA was mutated have aimed to elucidate the importance of particular domains, regions, and residues in CA functions. Those targeted mutagenesis studies largely relied upon insertion [53], deletion [54], or alanine or proline scanning [55], [56]. Here, we took a different approach to investigate the genetic robustness of HIV-1 CA. Specifically, we describe the generation of a large randomly mutagenized library of CA sequences to simulate the natural process of random mutation that occurs during HIV-1 replication. Strikingly, we find that CA is extremely intolerant of nonsynonymous substitutions, with ∼70% of random single nucleotide substitutions leading to a >50-fold reduction in replicative fitness. We also determined the biological basis for this extreme genetic fragility and found that requirements imposed by the need to accurately and efficiently assemble a mature virion are largely responsible. Indeed, a subset of mutants were temperature sensitive (ts), and the conditional non-viability of these mutants was always manifested during the formation of virions. Interestingly, fewer than half of the CA mutations that might be expected to occur *in vivo*, based on their high replicative fitness, were actually observed in natural populations. This finding suggests that CA sequence is constrained *in vivo*, not only by the need to maintain replicative fitness, but also by other unknown selective pressures. The mutational fragility of HIV-1 CA demonstrated herein is consistent with the relatively high overall degree of amino acid sequence conservation observed in natural populations, and potentially explains the apparently limited capacity of HIV-1 to evade immune responses directed against epitopes in CA.

## Results

### Experimental design and analysis

**Figure 1:**
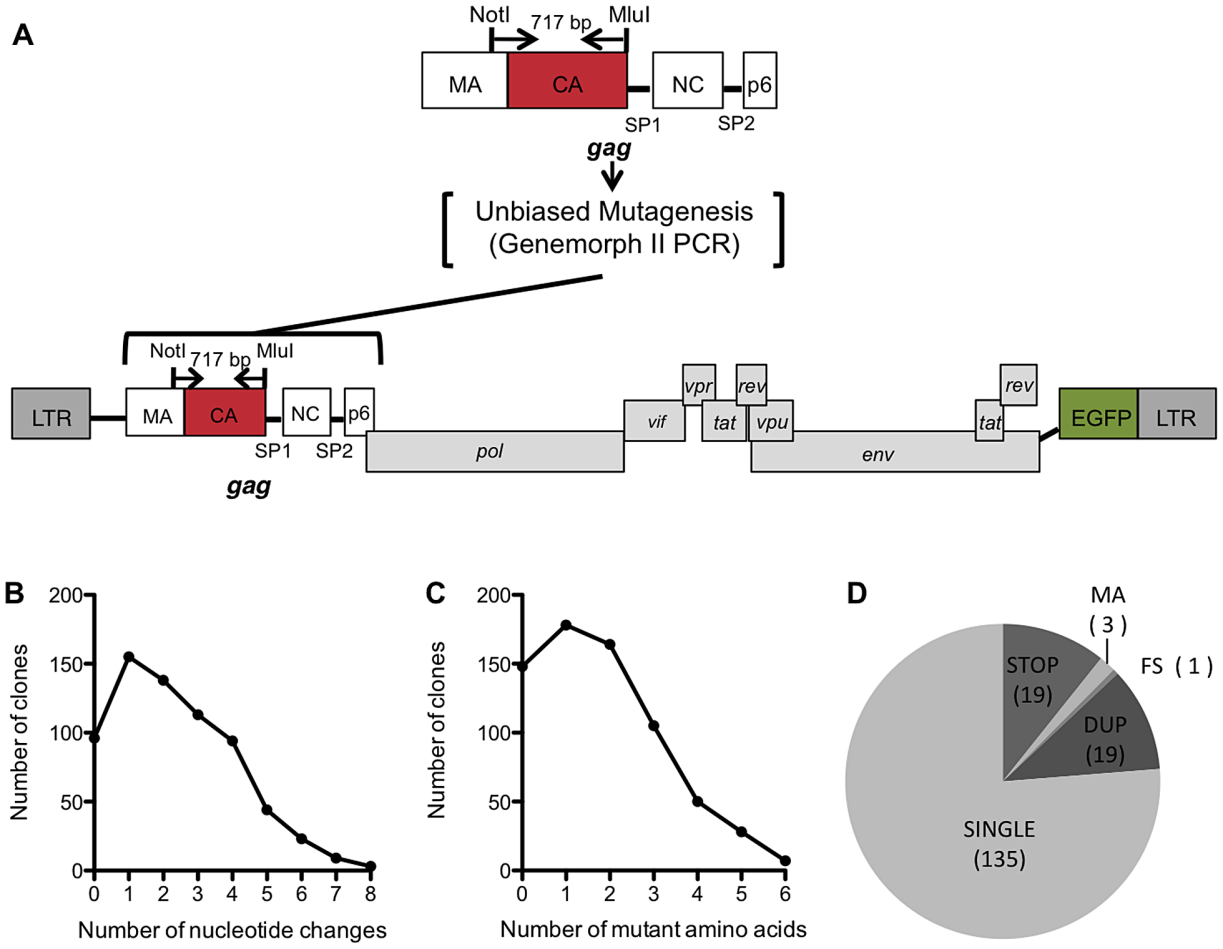
Generation and characterization of the CA mutant library. (A) Schematic representation of the replication competent HIV-1 proviral plasmid pNHGcapNM used in this study. NotI and MluI restriction sites flank a region of Gag that includes nearly all of CA, and a few amino acids in MA. This 717 bp sequence was subjected to Genemorph II mutagenesis and subcloned into pNHGcapNM. (B) Distribution of the number of nucleotide changes in each clone for the resulting library (this includes sequences that were represented more than once). (C) Distribution of the number of amino acid changes in each clone in the library (this includes sequences that were represented more than once, nonsense mutations, mutations in MA and frameshifts). (D) Pie chart describing all of the resulting single amino acid changes. SINGLE clones were those included in further studies of the library, and the remaining clones were discarded for the following reasons: STOP (mutation resulted in a stop codon), MA (contained a mutation in the small fragment of MA included in the amplicon), FS (frameshift), DUP (duplicate mutation of clone already in library).

To construct a library of random mutants of HIV-1 CA, a low fidelity PCR approach was used, as illustrated in Figure 1A. In this scheme, CA encoding sequences were amplified by error-prone PCR and cloned using a TOPO TA cloning Kit to generate a library with an estimated complexity of 1×10^4^ clones. Plasmid DNA extracted from the pooled library was then digested, and CA sequences inserted into a replication competent proviral clone encoding EGFP in place of Nef (pNHGcapNM accession:JQ686832, referred to as WT or parental virus hereafter) using unique *Not*I and *Mlu*I restriction sites, introduced by silent mutagenesis into sequences flanking those encoding CA (Figure 1A). Thereafter, nearly 1000 individual colonies were picked and proviral plasmid DNA was extracted from individual mini-cultures. The mutagenized CA sequence was then sequenced for each clone, and after failed sequencing reactions or chromatograms indicating the presence of more than one template were removed, the resulting library consisted of 680 arrayed proviral plasmids. The distribution of all nucleotide changes (Figure 1B), missense mutations (Figure 1C), and nonsense and other mutations (Figure 1D) within the library was then determined. The PCR-mutagenesis conditions were optimized in pilot experiments so as to reduce the representation of non-mutated and heavily mutated CA sequences in the library. Consequently, the most frequent mutation in the library was a single nucleotide or amino acid substitution (Figure 1B, C, D).

### Viability of a large panel of HIV-1 CA mutants

**Figure 2:**
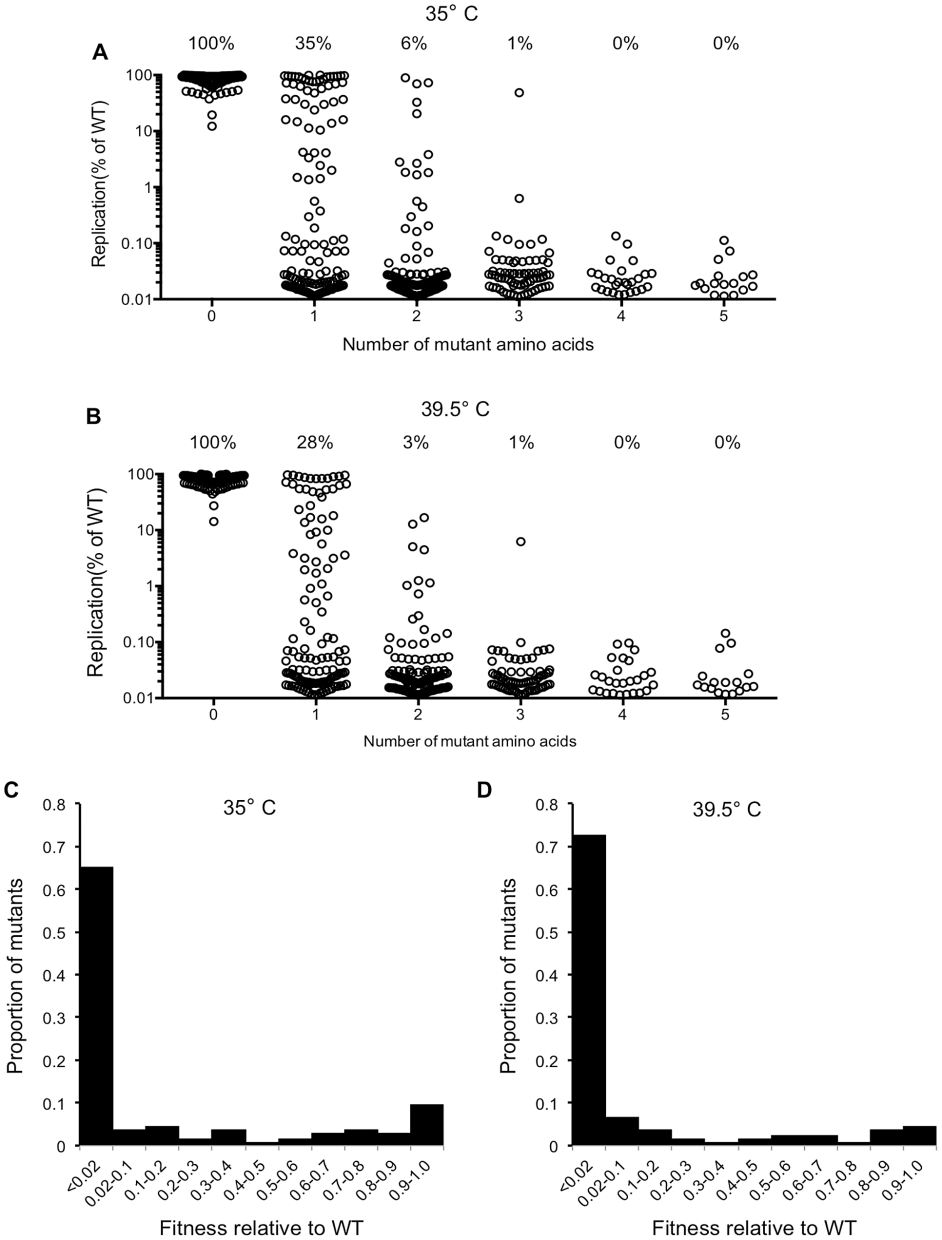
Fitness effects of mutations in the CA mutant library. (A) Viability of all CA mutant clones at 35°C (this excludes nonsense mutations, duplicates, frameshifts, MA mutations), demonstrating how the number of amino acid mutations in CA affects fitness (Replication is plotted as the fraction of MT-4 cells that are GFP+ (infected), as a percentage of the fraction infected by WT virus following a spreading replication assay). (B) Viability of all CA mutant clones at 39.5°C (this excludes nonsense mutations, duplicates, frameshifts, MA mutations), measured as in (A). (C) Distribution of mutational fitness effects (DMFE) for all single amino acid CA substitution mutants at 35°C. (D) DMFE for all single amino acid CA substitution mutants at 39.5°C.

To measure the genetic robustness of CA, we analyzed the ability of the 680 mutants to replicate. Specifically, replicative fitness was measured by means of a spreading infection assay, in which MT-4 cells were challenged with a low volume of virus-containing supernatant derived from transfected 293T cells (equivalent to an MOI of ∼0.01 for the WT virus). Multiple rounds of the virus replication cycle were allowed to occur before halting the experiment when the WT virus had infected >50% of cells (72 to 80 hours later). Replicative fitness for each CA mutant was expressed as number of cells that were infected (EGFP-positive), as a percentage of the number of cells that were infected by the WT virus (Figure 2A, B). Additionally, to allow for the isolation of temperature sensitive mutants that could facilitate the subsequent characterization of the replication defects associated with CA mutations (see below), this viability screen was carried out at two temperatures, 35°C (Figure 2A) and 39.5°C (Figure 2B).

Virtually all of the viral clones containing WT CA sequence, or only silent mutations, replicated at levels close to that of the starting construct. This indicated that the frequency with which defective viruses were generated by cloning artifacts or silent mutations was extremely low (Figure 2A, B). Moreover, this analysis revealed CA to be extremely fragile, or intolerant of randomly introduced amino acid substitutions (Figure 2A, B). If the cut-off for being viable is arbitrarily set at 2% of the parental virus replicative fitness, only 35% and 28% of the 135 clones that encoded single amino acid substitutions were viable at 35°C and 39.5°C respectively. These figures could overestimate the proportion of mutants that might be expected to be fit *in vivo*, as requirements for replication in an infected individual might be more stringent than in highly permissive MT-4 cells, and 2% of WT virus fitness in MT-4 cells is a rather generous cut-off figure for a designation of ‘viable’. Increases in the number of amino acid substitutions further decreased the frequency of viable mutants; only 6% and 3% of the 125 clones containing two random amino-acid substitutions were viable at 35°C and 39.5°C respectively. Only 1 of 73 clones containing 3 random amino acid substitutions was viable, and none of the 45 library clones with four or five random substitutions could replicate at all.

### Analysis of single amino acid CA mutants

In total, the subset of mutants that had a unique single amino acid substitution within CA (135 mutants) covered 102 (44%) of the 231 CA residues (for 33 CA residues there was more than one unique mutation at each position). To refine our estimate of CA robustness, two more assays were conducted at a natural temperature of 37°C, using only the panel of CA mutants that had unique single amino acid substitutions. First, in the MT-4 spreading assay, 40 mutant viruses (30%) exhibited at least 2% of parental virus fitness, and are listed in Table 1. Conversely, Table 2 contains the longer list of 95 mutants that had less than 2% of WT fitness in the spreading infectivity assay, and are considered non-viable. Single-cycle infectivity was also measured by transfecting 293T cells with proviral plasmids and measuring infectivity using a larger dose of virus (equivalent to an MOI ∼1 for the parental clone) and treating the MT-4 cells with dextran sulfate 16 h post-infection to limit replication to a single-cycle. Replicative fitness in spreading versus single-cycle assays was well correlated (Table 1), although most mutant viruses fared better in the single-cycle assay than in the spreading assay. This phenomenon possibly reflects the effect of transfection in the single-cycle assay, whereby overexpression of the viral proteins may suppress defects, as opposed to the spreading infection assay where more natural levels of viral gene expression are attained. Alternatively, the multiple rounds of replication in the spreading assay might amplify the effect of a defect in the single-cycle assay.

**Table 1:** Fitness measurements for viable HIV-1 CA mutants.

HIV CA mutant	Location in CA	Single cycle infectivity (% of WT)^a^	Spreading fitness (% of WT)^b^	Frequency in subtype B isolates (%)^c^
I2L	β-strand	90	66	0.1
N5D	β-strand	20	5	0.2
I6T	β-strand	52	57	0
M10I	β-strand	19	3	0.1
M10L	β-strand	74	27	0
M10V	β-strand	11	16	0.1
Q13H	β-strand	27	6	4.2
A14S	Loop	77	81	2.9
A14T	Loop	98	87	1.2
I15V	Loop	32	14	0.3
N21S	Helix 1	56	47	0
S33C	Loop	40	48	0
Q50H	Helix 3	20	3	5.3
E75D	Helix 4	41	22	5.8
V83M	Helix 4	75	65	2.6
H87Q	Cyclophilin b.l.*	120	92	13.7
H87R	Cyclophilin b.l.	28	2.5	0.2
I91T	Cyclophilin b.l.	85	77	0
I91V	Cyclophilin b.l.	100	82	41.3
M96I	Loop	120	92	11.6
E98D	Loop	101	63	5.1
R100S	Loop	54	43	0
S146C	Loop	44	34	0
T148I	Loop	66	37	0
S149C	Loop	65	74	0
S149G	Loop	71	30	0.2
I150V	Loop	19	3	0.2
I153T	Loop	21	5	0.4
Y164F	Helix 8	64	31	0
R167Q	Helix 8	11	6	0
A177S	Loop	99	37	3.0
M185I	Helix 9	24	3	0
E187V	Helix 9	60	47	0
L190M	Loop	38	33	0.1
T200S	Helix 10	104	90	1.6
A204G	Helix 10	77	89	8.7
G208E	Loop	15	4	0
A209T	Loop	108	94	0
A209V	Loop	72	84	0.1
T216A	Helix 11	72	38	3.0

a Infectivity measurement in which MT-4 cells were inoculated with supernatant from 293T cells (equivalent to MOI = 1 for WT virus) that had been transfected with single residue CA mutant proviral plasmids. Dextran sulfate was added approximately 16 h after infection to restrict replication to a single cycle. Values are reported as the percentage of the number of infected (GFP+) cells obtained with the WT virus.

b Fitness measurement in which MT-4 cells were inoculated with supernatant from 293T cells (equivalent to MOI = 0.01 for WT virus) that had been transfected with single residue CA mutant proviral plasmids. Multiple cycles of replication were permitted over 72 h. Values are reported as the percentage of the number of infected (GFP+) cells obtained with the WT virus.

c The frequency at which the indicated mutant residue occurs in 1000 HIV-1 subtype B sequences.

* Abbreviation for cyclophilin binding loop.

Mutants were considered viable if they maintained or 2% of WT fitness in the spreading replication assay.

**Table 2:** Non-viable HIV-1 CA mutants.

CA mutants in NTD	Location in CA	CA mutants in CTD	Location in CA
I2T	β-strand	I153M	Loop
H12R	β-strand	E159V	Loop
Q13R	β-strand	F161C	Helix 8
S16P	Loop	D163G	Helix 8
R18G	Helix 1	V165A	Helix 8
N21Y	Helix 1	F168S	Helix 8
K25I	Helix 1	R173I	Helix 8
V26E	Helix 1	A174G	Loop
K30E	Loop	K182Q	Helix 9
K30N	Loop	W184L	Helix 9
F32L	Loop	W184R	Helix 9
F32S	Loop	T186A	Helix 9
S33I	Loop	L190S	Loop
P34S	Helix 2	A194V	Loop
V36A	Helix 2	N195S	Loop
M39T	Helix 2	I201N	Helix 10
M39V	Helix 2	L211I	Helix 11
F40L	Helix 2	E212D	Helix 11
F40Y	Helix 2	M214L	Helix 11
L43I	Helix 2	M215V	Helix 11
G46R	Loop	A217V	Helix 11
P49L	Helix 3	Q219P	Loop
L52F	Helix 3	G220V	Loop
N57S	Helix 3	V221A	Loop
V59A	Loop	K227I	Loop
V59M	Loop	K227N	Loop
G60W	Loop	K227R	Loop
Q63R	Helix 4		
A65V	Helix 4		
M66I	Helix 4		
M66V	Helix 4		
M68L	Helix 4		
M68T	Helix 4		
L69I	Helix 4		
I73N	Helix 4		
A77D	Helix 4		
W80R	Helix 4		
P90T	Cyclophilin bl		
Q95L	Loop		
Q95R	Loop		
M96T	Loop		
P99A	Loop		
P99L	Loop		
R100W	Loop		
S102R	Helix 5		
A105G	Helix 5		
T110I	Loop		
L111F	Helix 6		
E113V	Helix 6		
I115K	Helix 6		
I115L	Helix 6		
I115T	Helix 6		
M118L	Helix 6		
M118V	Helix 6		
H120P	Loop		
N121I	Loop		
I124N	Loop		
V126A	Helix 7		
I129M	Helix 7		
I129T	Helix 7		
Y130C	Helix 7		
Y130H	Helix 7		
R132G	Helix 7		
G137E	Helix 7		
L138I	Helix 7		
M144V	Helix 7		
L151I	Loop		
L151Q	Loop		

Mutants were considered non-viable if they exhibited <2% of WT fitness in the spreading replication assay as described in Table 1.

Most of the viable CA mutants displayed attenuated infectivity, with 89% of single amino acid substitution causing a greater than 2-fold reduction in infectious virion yield. This suggests that it is difficult to improve upon the fitness of the parental CA, and that perhaps the parental CA sequence is close to a fitness peak. In previous analyses of panels of single amino acid mutants of RNA viruses, the distributions of mutational fitness effect (DMFE) values were bimodal in nature, with substitutions causing either lethality, or only minor decreases in fitness [4]–[9]. This phenomenon was observed to some extent for HIV-1 CA (Figure 2A–D). However, the proportion of viable mutants was significantly smaller than has been observed in previous studies [4]–[9], and the selective occurrence of mutants with only minor fitness defects was not prominent (Figure 2A–D).

**Figure 3:**
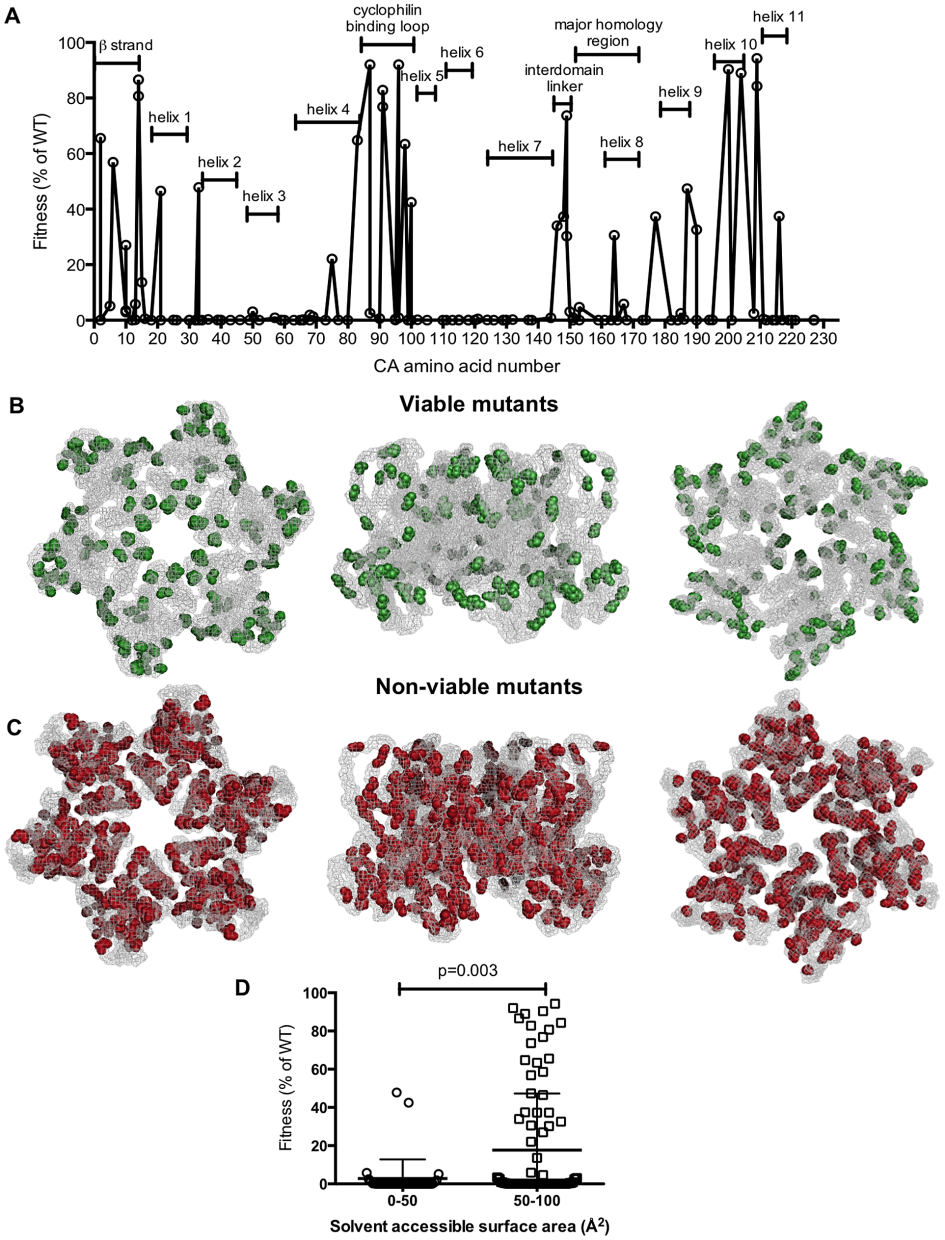
Distribution of single amino acid substitutions in CA and their effects on fitness. (A) Plot of HIV-1 fitness values for CA mutants with the location of the mutation arranged on the X-axis from left (N-terminal residue) to right (C-terminal residue), and their corresponding fitness (as a % of WT in a spreading replication assay) as described in Tables 1 and 2. (B, C) Location of single amino acid mutations in an X-ray crystal structure of the CA hexamer (PDB: 3GV2). Leftmost images show the hexamer viewed from the exterior of the intact capsid, center images show the hexamer viewed from within the plane of the capsid lattice and rightmost images show the hexamer viewed from the interior of the intact conical capsid. Residues that gave greater than 2% of WT fitness in a spreading replication assay when mutated (viable mutants) are shown in green (B). Residues that gave less than 2% of WT fitness in a spreading replication assay when mutated (non-viable mutants) are shown in red (C). (D) Comparison of fitness and residue exposure, using solvent accessible surface area (determined using UCSF Chimera with PDB 3GV2). Mutated residues were placed in two groups that had either (i) a solvent accessible surface area <50 Å^2^ or (ii) a solvent accessible surface area >50 Å^2^. The corresponding fitness measurement for each mutant is plotted.

A comparison of the Tables 1 and 2 reveals that different mutations at certain positions in CA, such as I2, can result in markedly different fitness effects, suggesting that the type of residue change may, in some cases, be of some importance in determining fitness. However, inspection Tables 1 and 2 indicates that this was not typically the case, and the position within CA rather than the type of residue change appeared generally more important in determining the effect of mutations on fitness. Of the 231 amino acids that comprise CA, 56.3% lie within CA's 11 helices, 5.6% are in the N-terminal β-strand, 3.9% are in the cyclophilin A binding loop, and the remaining 34.2% are in other inter-helical ‘loops’. These numbers can be used to provide context to the summary of mutant fitness values to indicate regions of particular genetic fragility in CA (Table 3, Figure 3A). Notably, the N-terminal β-strand, the cyclophilin binding loop, and interdomain linker appeared comparatively robust, with 70–100% of mutants in these regions retaining some viability. In contrast, helices were less tolerant of mutation, with a mere 16% of mutants in these domains retaining some viability, while the interhelical loops, where 39% of mutants retained viability, had intermediate robustness. Helices 2, 5, 6, and 7 were particularly fragile, with all 25 mutations in these sequences causing non-viability. A graphical representation of these results, where the fitness of each mutant is plotted against its position in the linear CA sequence (Figure 3A), confirms that regions of mutational fragility are non-uniformly distributed in CA, with mutations in NTD helices being extremely likely to cause inactivation (Figure 3A).

**Table 3:** Summary of CA mutant viability by CA region.

Region	Number of viable mutants	Number of non-viable mutants	% Viable mutants
β-strand	7	3	70
Helix 1	1	4	20
Helix 2	0	7	0
Helix 3	1	3	25
Helix 4	2	10	17
Cyclophilin b.l.	4	1	80
Helix 5	0	2	0
Helix 6	0	7	0
Helix 7	0	9	0
Helix 8	2	5	29
Helix 9	2	4	33
Helix 10	2	1	67
Helix 11	1	5	17
MHR	3	6	33
Interdomain linker	5	0	100
Helices (all)	11	57	16
Loops (all)	22	35	39
NTD	27	68	28
CTD	13	27	33

When displayed in the context of a capsid hexamer structure (PDB: 3GV2, Figures 3B, C), amino acids corresponding to viable mutants (in green, Figure 3B) appeared to occur preferentially in surface exposed residues. Conversely, mutations in the interior of the CA structure almost always generated non-viable mutants (displayed in red, Figure 3C). This finding is particularly evident when the CA hexamer is viewed from the point of view of interior of the assembled capsid (Figure 3B,C, rightmost diagrams), and reinforces the impression of an inner CA structure, or ‘core,’ composed of helices that are particularly sensitive to mutation.

Analysis of the solvent accessible surface area for individual mutated residues confirmed these findings. Specifically, mutated residues with solvent accessible surface areas of less than 50 Å^2^ (an appropriate cutoff for surface exposure based on previous mutagenesis analyses [57]) caused significantly greater fitness defects (p = 0.003). Viruses with such mutations and exhibited a mean fitness value of 2.8% of WT. Conversely, viruses harboring mutations of more surface exposed amino acids (solvent accessible areas of greater than 50 Å^2^) exhibited a mean fitness value of 18% of WT.

### Biological basis for genetic fragility in HIV-1 CA

As outlined in the introduction, CA performs a number of functions in HIV-1 replication, including mediating the assembly of immature virions (in the context of the Gag precursor) and formation of a mature conical capsid. Moreover, a capsid of optimal stability is thought to be important during the uncoating step of the HIV-1 cycle in newly infected cells, and CA is also key for the import of the viral genome into the nucleus of newly infected cells. To determine which of these functions, if any, contributed the genetic fragility of HIV-1 CA, we performed a number of assays to elucidate the nature of the replication defects in constitutively or conditionally non-viable CA mutants.

To facilitate the elucidation of the nature of the replication defects in CA mutants, we first focused on the subset of conditionally non-viable, ts mutants. At outlined in Figure 2, initial determinations of mutant fitness were carried out at 35°C and at 39.5°C. While most CA mutants were approximately equally fit or unfit at both temperatures, a few exhibited a ts phenotype that was quite large (Figure S1). Some of the ts mutants (circled in Figure S1 and listed in the materials and methods) included double mutants. We therefore generated CA mutants that encoded each single amino acid substitution in isolation. Ultimately, this yielded eleven single amino-acid CA mutants with substantial ts replication phenotypes.

**Figure 4:**
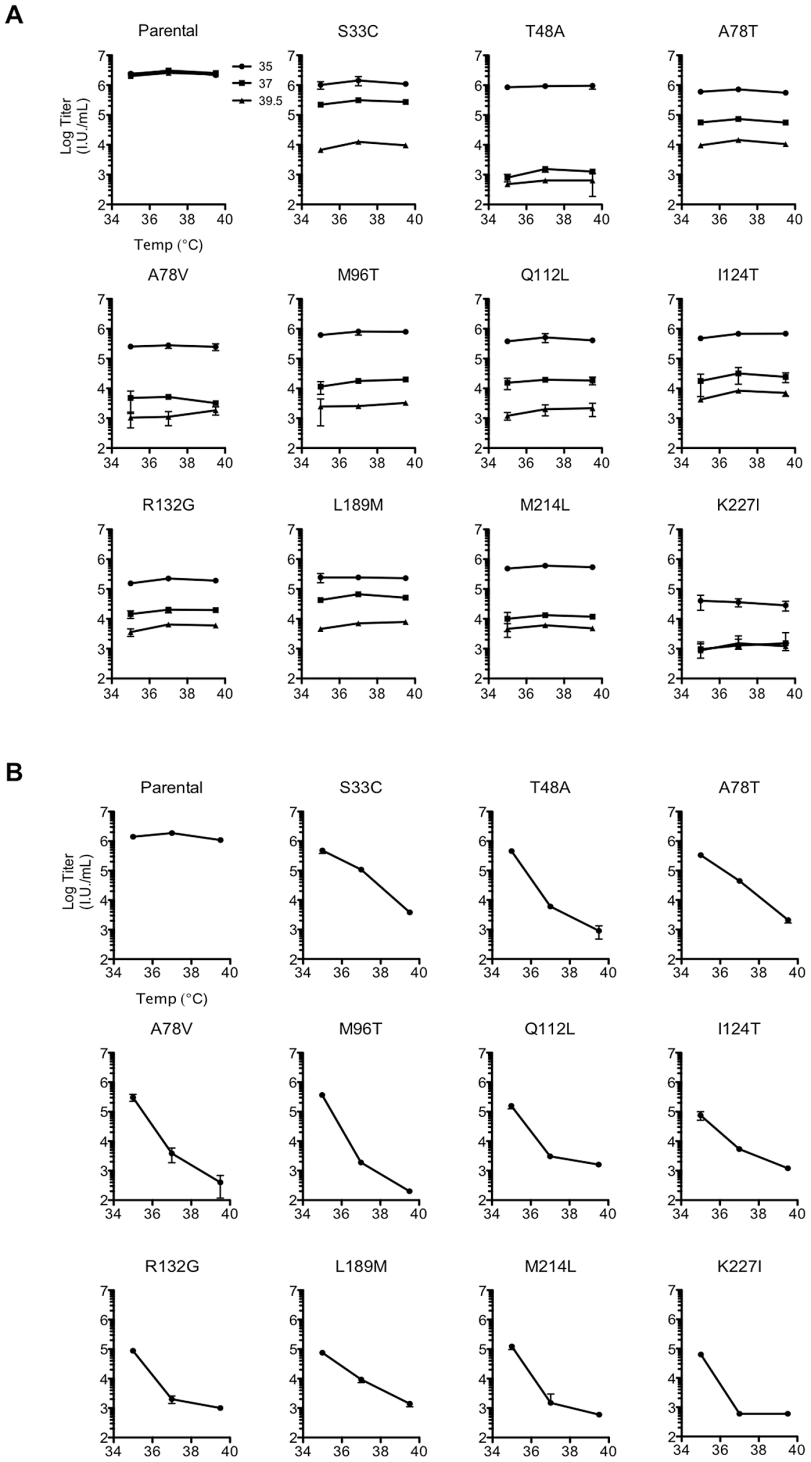
Conditionally viable (ts) CA mutants exhibit defects only when assembled at the non-permissive temperature. (A) Measurement of infectious virion titer from 293T cells transfected with either the parental or the ts single residue mutant proviral plasmids, and placed at either 35°C (filled circles), 37°C (filled squares), or 39.5°C for virion production (filled triangles), as indicated by distinct plot lines. The titer of infectious virus in the resulting supernatants from each mutant was measured in MT-4 cells at 35, 37, or 39.5°C as indicated on the x-axis, with the addition of dextran sulfate at approximately 16 h post-infection to limit replication to a single cycle. Note that some single ts mutants were resolved from double or triple mutants (see Materials and Methods) so do not appear in Table 1 or 2. (B) As in (A), however, here the temperature of virus production is indicated on the x-axis, and inoculations were done in MT-4 cells at 37°C only.

Because the initial screen measured replicative fitness over multiple rounds of replication, it could not determine what specific step in the viral life cycle was impaired in the constitutively or conditionally non-viable mutants. We therefore determined the single-cycle infectivity of the ts CA mutants using virions generated in 293T cells at 35, 37, or 39.5°C, which were then used to infect MT-4 cells at 35, 37, or 39.5°C (Figure 4A, B). Importantly, the WT control, pNHGcapNM, maintained a consistent viral titer regardless of production and infection temperatures (Figure 4). Most of the ts CA mutants had similar, or modestly reduced, infectivity as compared to the parental virus when virion production and infection was done at the permissive temperature (35°C). Strikingly however, single-cycle replication of all the ts mutants was inhibited at least 50 to 1000-fold when restrictive temperatures were applied during virion production (Figure 4A, B). Conversely, all of the ts mutants were completely unaffected when restrictive temperatures were applied only during infection and not during production (Figure 4A, B). This result indicated that the defects associated with conditionally non-viable ts CA mutants occurred exclusively and irreversibly, during or shortly after, particle production, and not during early steps of the viral life cycle (e.g. uncoating or nuclear import).

**Figure 5:**
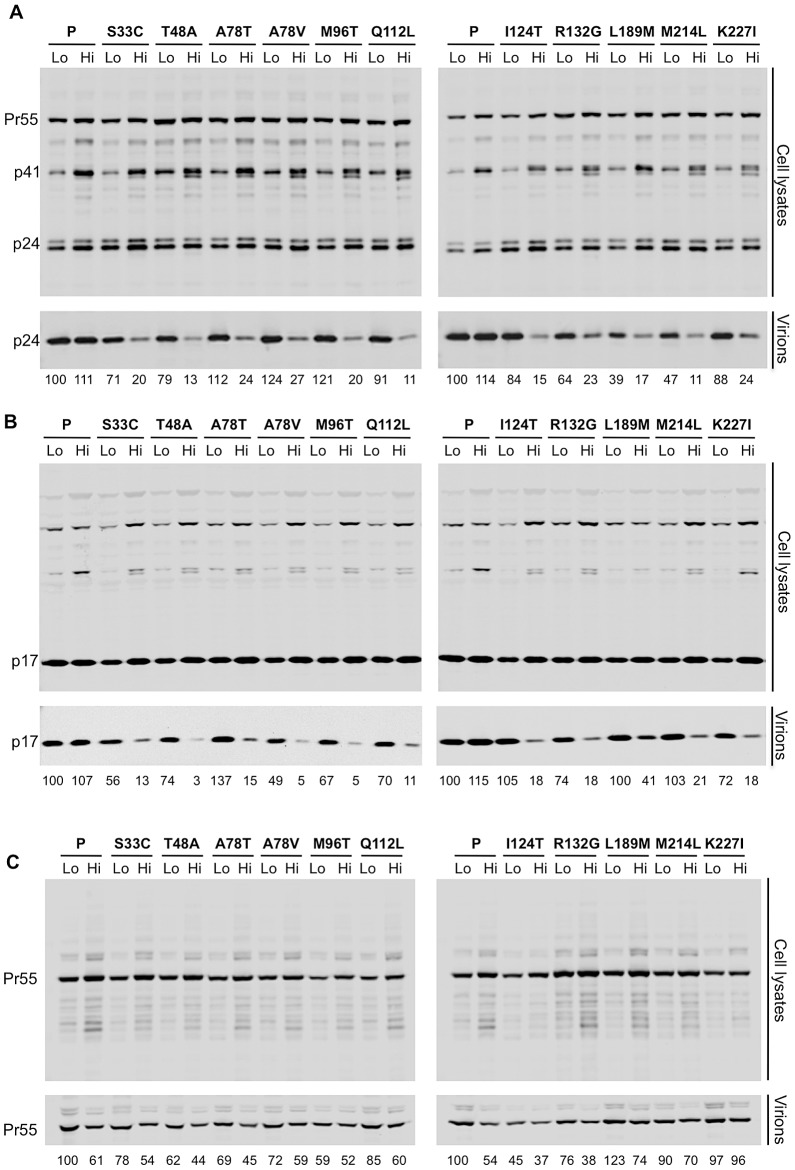
All ts CA mutants exhibit protease-dependent reduction in extracellular particle yield at the non-permissive temperature. (A). Western blot analysis (using an anti-CA antibody) of cell lysates and virions generated by transfected 293T cells. Two lanes are shown for each mutant: on the left is the sample from cells that were incubated at 35°C following transfection (Lo) and on the right is the sample from cells that were incubated at 39.5°C following transfection (Hi). Numbers below each lane indicate fluorescence intensities (LiCOR) associated with the CA protein pelleted from virion-containing supernatant. (B) Western blot analysis using an anti-HIV-1 MA antibody (p17) of cell lysates and virions, for the same panel as shown in A. Numbers below each lane indicate fluorescence intensities (LiCOR) associated with the MA protein pelleted from virion-containing supernatant. (C) Western blot analysis for the same panel of ts CA mutants as in A, but expressed in the context of a protease-defective proviral plasmid. Numbers below each lane indicate fluorescence intensities (LiCOR) associated with the CA protein pelleted from virion-containing supernatant.

Analysis of Gag expression and processing by the WT virus and the ts CA mutants revealed that similar levels of cell-associated Gag precursor Pr55 and capsid p24 were present regardless of the production temperature (Figure 5A). However, unlike the WT control, each of the ts CA mutant viruses generated 57% to 88% less extracellular virion-associated p24 at the restrictive temperature (Figure 5A). Because it was possible that the mutations could have affected the CA protein stability or recognition by the p24 antibody, we also analyzed Gag expression, processing and particle generation by the ts mutants using an anti-p17 matrix (MA) antibody (Figure 5B). This analysis yielded similar results. Specifically, there were approximately uniform levels of p17 in cell lysates for each of the ts CA mutants regardless of temperature. However, reductions in extracellular particulate p17 protein of 2.4–25-fold indicated that particle production was decreased by 59–96% for the ts mutants at the nonpermissive temperature.

Although the 2.3 to 25-fold reduction in particle production observed for the ts CA mutants is smaller than the corresponding 50 to 1000-fold reductions in infectious virion yield, it is important to note that the majority of this reduction in infectivity (specifically 57% to 96%) was due to the inability of these mutants to efficiently generate particles. However, because the block in the generation of extracellular particles was not absolute, and there was residual generation of poorly infectious particles, it appeared that particle formation by the ts mutants was attenuated to varying degrees at the non-permissive temperature rather being than completely defective. The inability of the ts mutants to efficiently generate virions at the restrictive temperature is entirely consistent with the finding that it was the temperature during virion production, not inoculation, that determined the phenotype of all of the ts mutants we identified, and that all these mutations conferred defects that are manifested during virion morphogenesis.

Careful inspection of both anti-CA and anti-MA blots of the ts mutants at the nonpermissive temperature revealed a slight abnormality in Gag processing. The parental virus appeared to generate a single ∼41 kDa band that reacted with anti-CA and anti-MA at both permissive and restrictive temperatures (presumptively the p41 MA-CA-p2 processing intermediate, Figures 5A and 5B). Conversely, one or sometimes two additional protein species, of similar but not identical mobility to p41 were observed for each of the ts mutants, specifically at the restrictive temperature. Thus, for each of the ts CA mutants, attenuated particle formation was accompanied by perturbation of Gag processing, which may have contributed to the overall defects in particle yield and infectiousness. Notably, eliminating viral protease activity eliminated the temperature-induced reduction in particle yield for all eleven ts CA mutants (Figure 5C). Thus, capsid mutations that caused temperature-dependent attenuation of particle formation do not do so prior to protease activation. This being so, and because inoculation temperature did not affect infectivity, it appeared that all eleven conditionally non-viable CA mutants have defects that are manifested during, and not before or after, virion morphogenesis.

### Most constitutively non-viable CA mutants exhibit attenuated particle formation

**Figure 6:**
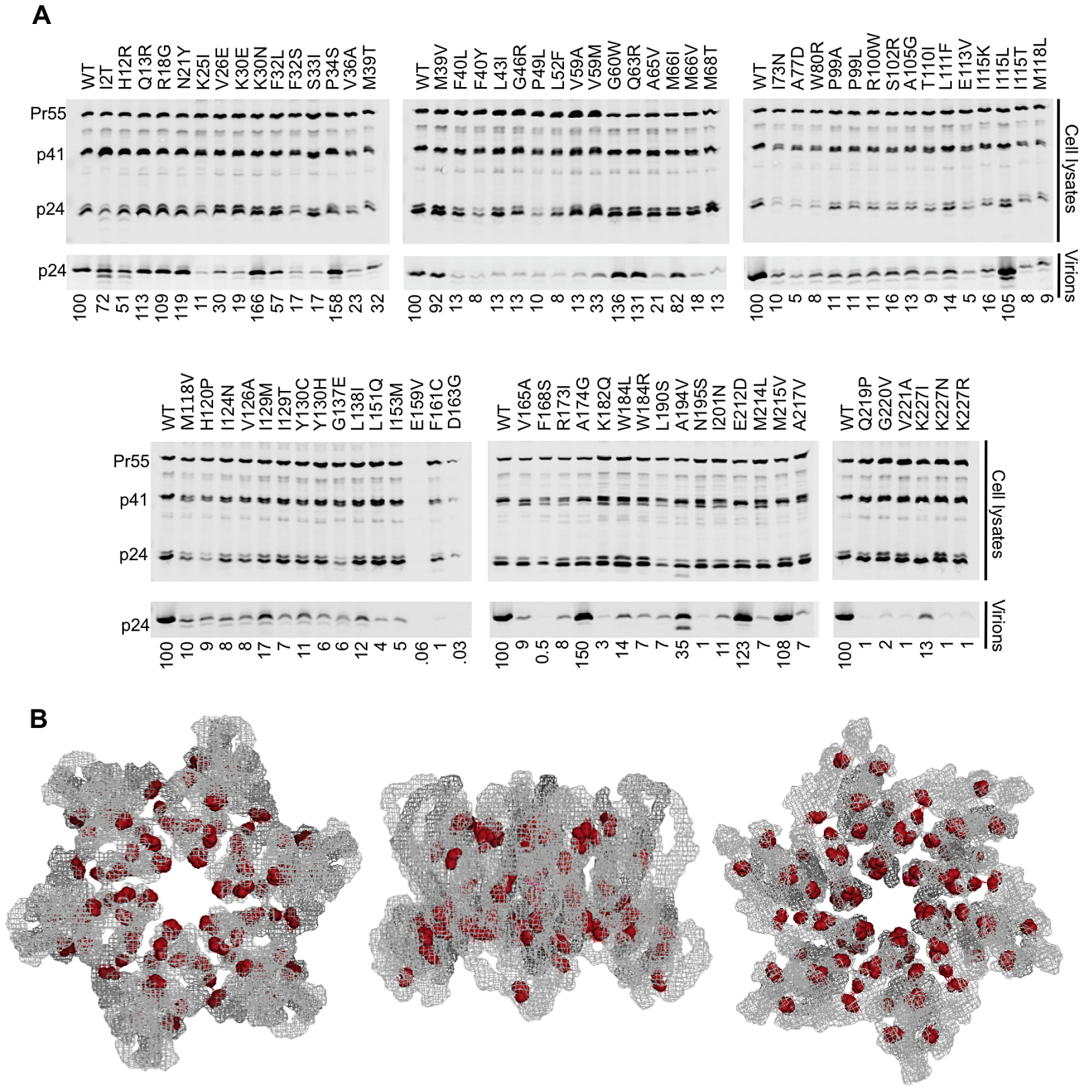
Most non-infectious constitutively non-viable CA mutants exhibit attenuated particle formation. (A) Western blots, probed with an anti-CA antibody, of cell lysates and virions for all constitutively non-viable CA mutants (those exhibiting <2% of WT fitness in a spreading replication assay). It should be noted that multiple substitutions between residues 159 and 168 resulted in diminished signal using the monoclonal anti-CA antibody (derived from hybridoma 183-H12-5C). Indeed CA carrying an R167Q mutation is infectious, but is not recognised by this antibody (unpublished observations) indicating that the epitope of the antibody lies in this region. This suggests that the E159V substitution (Figure 6A) might also disrupt antigen recognition, accounting for the absence of signal in this lane. (B) Location of mutations in the CA hexamer that are constitutively non-viable but exhibit near WT levels of particle formation. The leftmost image shows the hexamer viewed from the exterior of the intact conical capsid, the center image shows the hexamer viewed from within the plane of the conical capsid lattice and the rightmost image shows the hexamer viewed from the interior of the intact conical capsid.

The above analysis of the eleven conditionally non-viable CA mutants suggested that requirements imposed during particle production, and not during any other phase of the viral life cycle, are responsible for the mutational fragility of HIV-1 CA. However, it was possible that the selection of conditionally rather than constitutively non-viable mutants could have biased this conclusion. Thus, we examined the ability of the larger set of 81 constitutively non-viable mutants to generate extracellular particles, using western blot assays (Figure 6A).

Most (74%) of the constitutively non-viable mutants exhibited a greater than 5-fold reduction in the yield of extracellular particles. A minority (20%) of these lethal mutations did not affect the magnitude of particle production (less than 2-fold changes in particle yield), but instead resulted in the efficient generation of non-infectious particles (Figure 6C). The remaining 6% of mutants gave intermediate phenotype with 2- to 5-fold reductions in particle yield. In a few instances, for example at residues K30, M39, and I115, a change to one amino acid resulted in efficient generation of extracellular particles that were non-infectious, while a change to another amino acid resulted in attenuated particle production. Nonetheless, there did not appear to be any general trend in changes of amino acid properties that might cause mutants to efficiently generate non-infectious virions as opposed to being attenuated for particle generation. Nearly all of the mutants that yielded WT levels of non-infectious particles mapped to the inner core of the hexamer, in a manner that was similar to the general distribution of inactivating CA mutations (compare Figures 6B and 3C). Of note, many constitutively non-viable mutants displayed evidence of perturbed processing, with the generation of additional ∼40 kDa Gag protein species, similar to observations with the conditionally non-viable mutants at the restrictive temperature (Figure 5 A, B). Overall, these data reinforce the conclusion that the primary underlying cause of mutational fragility in HIV-1 CA is the need to mediate accurate and efficient particle assembly.

### Reduced particle formation in most conditionally and constitutively non-viable CA mutants

**Figure 7:**
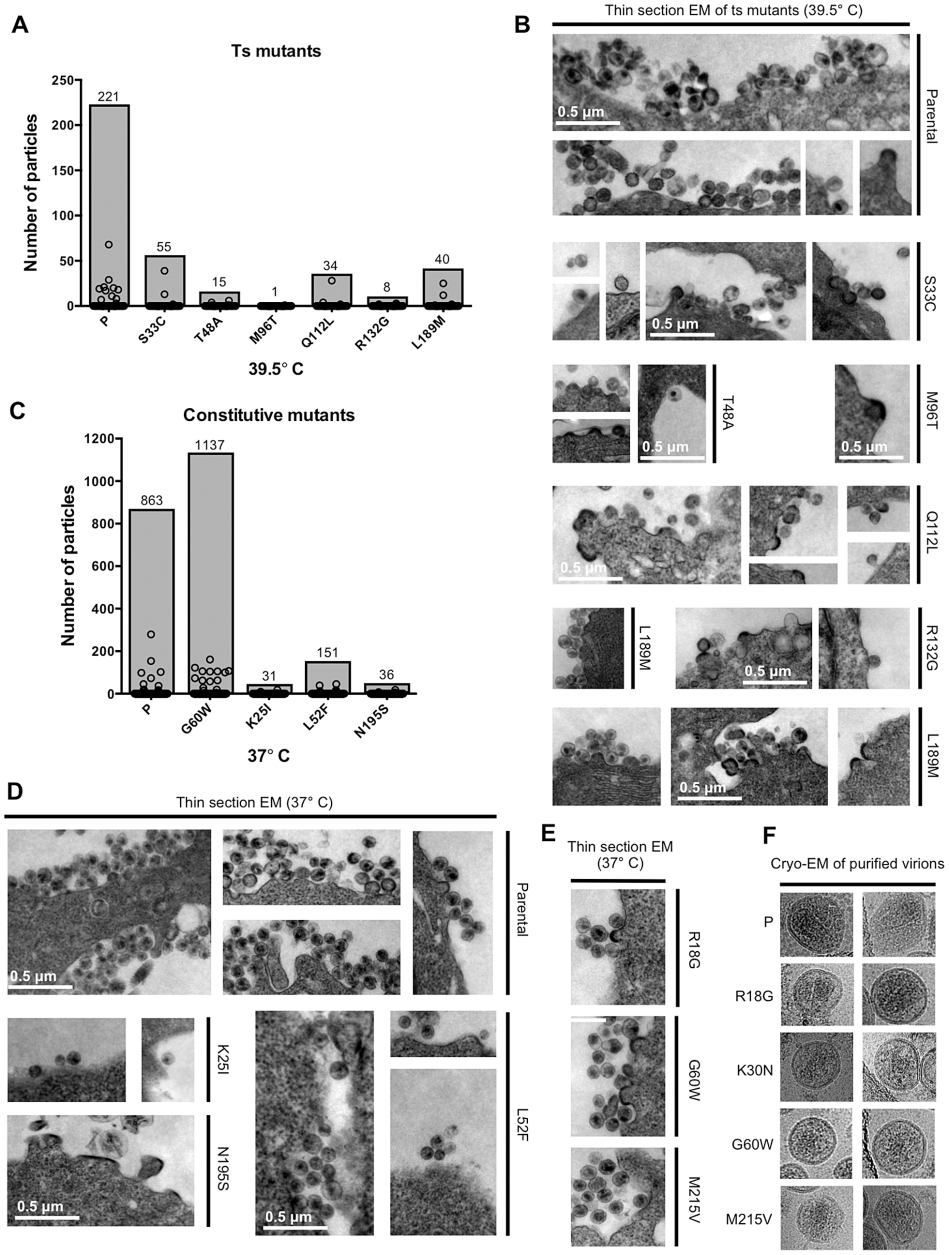
Electron microscopic analysis of particle formation by selected conditionally and constitutively non-viable CA mutants. (A) Analysis of thin-section EM images showing parental virus and ts CA mutant particle production at the restrictive temperature (39.5°C) in 293T cells. Circles represent the number of particles associated with individual cells (n = 150 for each sample), while shaded bars demonstrate the total number of particles counted in the150 cells. (B) Representative images of the thin-section EM samples analysed in (A). Scale bars shown apply to all images. (C) Analysis of thin-section EM images, as in A, showing parental virus and constitutively non-viable CA mutant particle formation at 37°C. Particles associated with 150 cells for each sample were counted and plotted as in A. (D) Representative images of the thin-section EM samples analysed in (C). Scale bars shown apply to all images. (E) Representative thin-section EM images for three constitutively non-viable CA mutants that yielded high levels of non-infectious particles. (F) Cryo-EM images of purified virions from parental virus and four constitutively non-viable CA mutants that yielded high levels of non-infectious particles.

To further examine the nature of the defects in conditionally and constitutively non-viable CA mutants, we employed electron microscopy (EM) to quantify and visualize particle morphogenesis. To examine the ts CA mutant mutants, 293T cells (at 39.5°C) were transfected with the parental or one of six randomly selected ts CA mutant proviral plasmids, plus a plamid expressing a modified Vpu-resistant human tetherin (delGI, T45I) [58] to retain virions at the plasma membrane and facilitate their visualization. Inspection of 150 cells for each ts CA mutant clearly showed these mutants generate far fewer total particles at the restrictive temperature (Figure 7A shaded bars), with reductions ranging from 4- to 221-fold (75–99.5% fewer particles). Some, but not all particles generated by the ts CA mutants appeared morphologically normal, but there appeared to be a higher fraction of abnormal particles and budding structures as compared to the parental virus (Figure 7B). However, the rarity with which the ts CA mutants generated discernable viral structures made it difficult to reach definitive conclusions about the morphological accuracy (or otherwise) of the particles that were formed.

We also performed thin-section EM analyses of cells transfected with three randomly selected constitutively non-viable mutants (K25I, L52F, and N195S) that exhibited reduced extracellular particle yield (Figure 7C,D). Again, this was done in the presence of tetherin (delGI, T45I) to facilitate particle imaging. This analysis revealed reductions in particle formation that were similar in character to those observed for the conditional ts CA mutants. Specifically, particle formation for these mutants was reduced ∼6 to 28-fold (82–96% fewer particles) as compared to the parental virus. The small number of particles that were generated by the K25I and L52F mutants appeared morphologically similar to the parental virus, while the rare budding structures generated by the N195S mutant were obviously irregular (Figure 7D). Interestingly, defects similar to that exhibited by N195S have been previously reported for CA mutations made at a nearby residue (D197) [56].

As an additional control for these experiments, particle formation by a constitutively non-viable mutant (G60W) that gave high levels of non-infectious particulate p24 in the western blot assay was quantified by EM analysis. In fact, the G60W mutant generated particles that were as, or even more, numerous than the parental virus. Overall, the data acquired via EM analysis of particle formation broadly corroborated the estimates of particle formation efficiency obtained using western blot assays (Figures 5A, 5B and 6A), and reinforce the notion that most randomly introduced CA mutations induced substantial attenuation, but not complete abolition, of particle formation.

Because a fraction (20%) of the constitutively non-viable CA mutants generated normal levels of extracellular particles that were non-infectious, we randomly selected three such mutants, (R18G, G60W, and M215V) for examination by thin-section EM (Figure 7E). Each of these mutants exhibited morphologies that were indistinguishable from the WT virus. Additionally, cell free virions produced by these mutants, plus one additional non-viable mutant (K30N) that also exhibited efficient particle formation, were also examined by cryo-electron microscopy (Figure 7F). This analysis again revealed particle morphologies that were similar to WT, with the possible exception of G60W, whose cores were more difficult to discern.

### Only a small fraction of randomly introduced CA mutants affect HIV-1 infection of non-dividing cells

**Figure 8:**
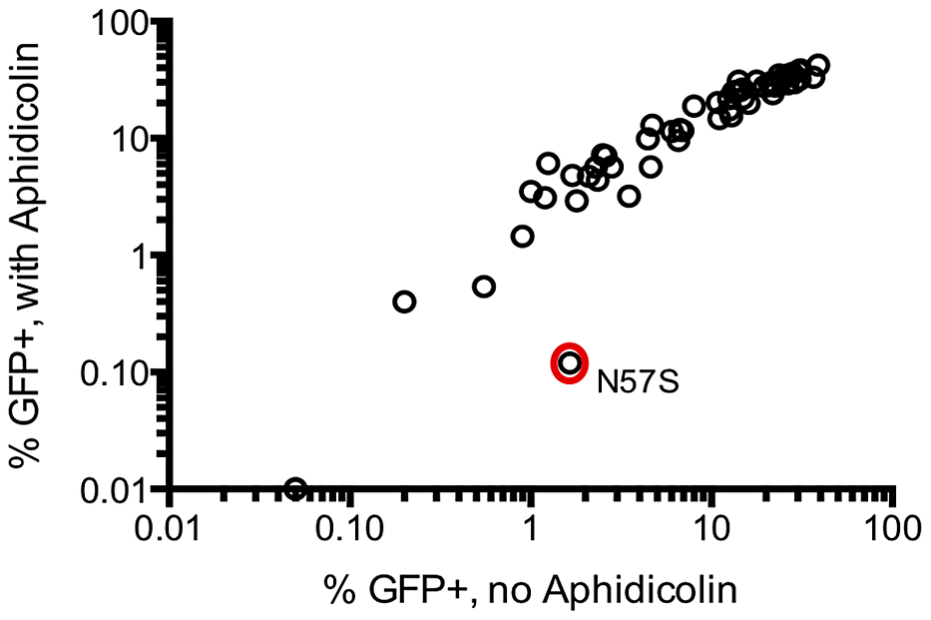
Most CA mutants do not exhibit cell cycle dependent infectivity defects. The infectivity of 58 CA mutants exhibiting readily measurable infectivity in a single cycle assay was measured using untreated TZM-bl target cells (x-axis), and following arrest of the cell cycle with aphidicolin (arrests cell cycle in the S-phase) at 2 µg/ml (y-axis).

The above data demonstrates that a large fraction of randomly introduced mutations impair the ability of HIV-1 CA to support efficient and accurate virion morphogenesis. To determine whether a similarly large fraction of CA mutants affected another critical function of CA, namely infection of non-dividing cells [31], we screened all CA mutants that exhibited easily measurable infectivity in single-cycle infection assays for their ability to infect cells in the presence or absence of aphidicolin (which arrests the cell cycle in S-phase). Only one of the 58 mutant viruses tested, N57S, exhibited substantially higher infectivity in the absence than in the presence of 2 µg/ml aphidicolin (Figure 8). A mutation at this position, N57A, has previously been reported to impose cell cycle dependence on HIV-1 infection [30]. Because only one of the 58 randomly introduced mutations tested affected the cell cycle independence of HIV-1 infection, these data suggest that CA sequence is constrained to a large extent by the need to accurately and efficiently assemble into functional viral particles, but to only a small extent by another key function, i.e. the need to infect non-dividing cells.

### Comparison of naturally occurring and randomly introduced CA mutations

**Figure 9:**
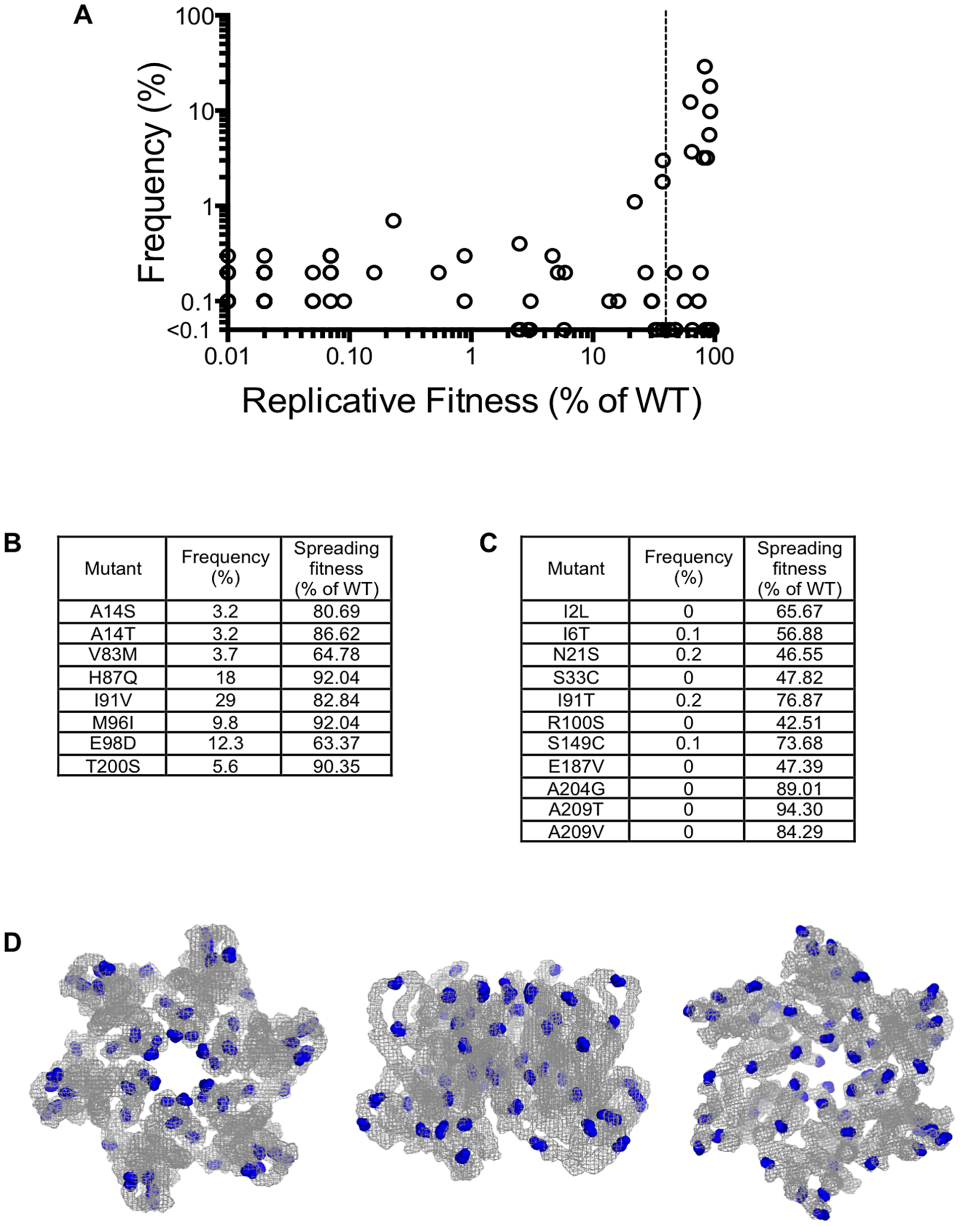
Occurrence of CA mutations in natural HIV-1 subtype B populations. (A) Plot of the frequency with which the library mutations occur in 1000 natural HIV-1 subtype B CA sequences (Y-axis) versus the fitness of the same mutations in a spreading replication assay (X-axis). The vertical dashed line indicates 40% of WT replicative fitness, below which mutants rarely occurred in nature. Mutants that exhibited less than 0.01% of WT infectivity, and were never observed in nature would appear at the origin of this graph and are not plotted. (B) List of the library mutants that occurred at a frequency greater than 3% in 1000 subtype B isolates, and their corresponding fitness values. (C) List of the library mutants with greater than 40% of WT fitness that occurred infrequently (<0.3%) or never in 1000 subtype B sequences, and their corresponding fitness values. (D). Location of residues in the CA hexamer where mutations resulted in minimal fitness costs (>40% of WT replication) but that occurred in less than 0.3% of subtype B isolates. The leftmost image shows the hexamer viewed from the exterior of the intact conical capsid, the center image show the hexamer viewed from within the plane of the capsid lattice and the rightmost image shows the hexamer viewed from the interior of the intact conical capsid.

Our measurements of *in vitro* fitness of HIV-1 CA mutants suggested that fitness should place tight constraints on the sequences of CA that are found in natural populations. Therefore, to compare the fitness of single amino acid HIV-1 CA mutants with their occurrence *in vivo*, we obtained a set of 1,000 CA sequences isolated from HIV-1 subtype B infected individuals, and determined the frequency with which mutations in our random mutant library were found in these naturally occurring sequences. A plot of the frequency with which CA mutations occur in natural populations against their measured *in vitro* fitness (Figure 9A) revealed that only mutants that exhibited a fitness of at least 40% of the WT CA sequence in our *in vitro* assays occurred at a frequency greater than 1% in natural populations. Curiously, however, the nineteen random mutants that exhibited >40% of parental virus fitness had bimodal distribution of occurrence *in vivo*. Some occurred relatively frequently (i.e. in >3% of natural sequences Figure 9B), while others occurred extremely rarely (<0.3% of natural sequences Figure 9C). That is to say that a subset of mutations that incur little or no apparent fitness cost appear to be largely absent from natural populations of subtype B HIV-1. Consistent with their lack of effect on fitness, virtually all of these apparently innocuous, but rare, mutations occurred on the outer surfaces of the hexamer, and avoided the inner hexamer core (Figure 9D).

### CA mutations that exhibit high fitness *in vitro* but occur rarely *in vivo* do not exhibit replication defects in primary cells

The existence of a subset of mutations that gave high fitness in MT-4 cells *in vitro*, yet were virtually absent in natural populations, suggested that some mutations may be selected against *in vivo* in a manner that was not revealed by our fitness assays. Previous studies have demonstrated that CA mutations can have cell-type dependent effects on HIV-1 infectiousness [38], [39], [59], [60], and it was therefore possible that these mutants might exhibit fitness defects that are manifested in natural target cells, but not in MT-4 cells. Thus, we performed replication assays in primary cell types using 8 apparently fit viruses containing mutations that occurred rarely in natural populations (Figure 9C) and 5 randomly chosen viruses containing mutations that occurred frequently (Figure 9B).

**Figure 10:**
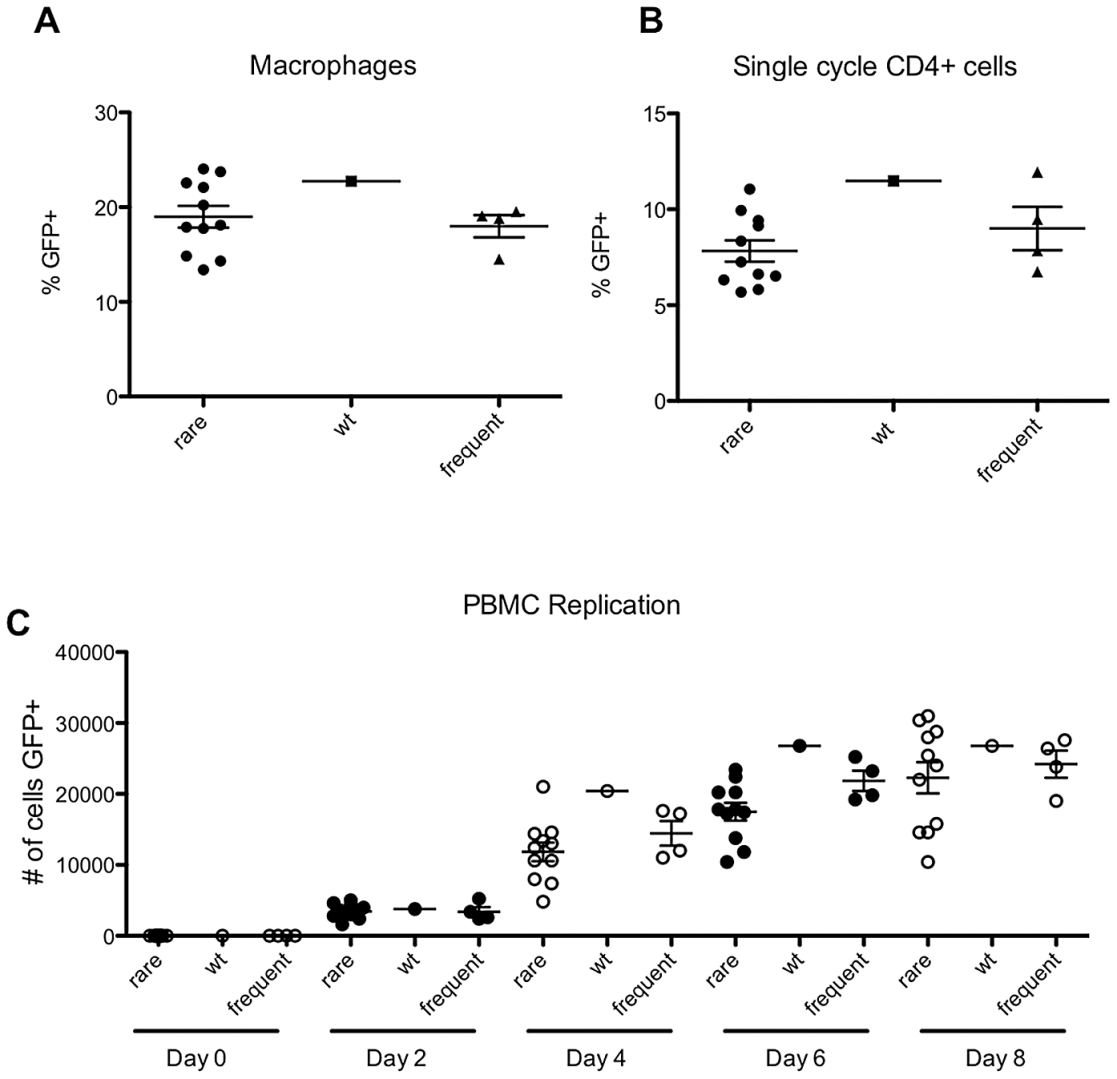
‘Rare but fit’ mutants behave indistinguishably from frequently occurring mutants in primary cells. (A) Percentage of infected macrophages following infection with VSV-G pseudotyped WT virus (NHGcapNM, filled squares) or derivatives containing mutations that occurred either frequently (filled triangles) or rarely (filled circles) in natural sequences, as indicated. (B) Percentage of infected stimulated CD4+ T cells, as indicated. (C) Spreading replication, following infection of stimulated peripheral blood mononuclear cells with WT virus or derivatives containing mutations that occurred either frequently or rarely in natural sequences, as indicated by the number of infected (GFP+) cells at the indicated times, after infection at an MOI of 0.1.

In fact, there was no significant difference between the ability of the rarely occurring or frequently occurring CA-mutant viruses to infect PBMC, primary CD4+ T cells or macrophages in short term (quasi single-cycle) infection assays (Figure 10A, B, C). Furthermore, there was no difference in the capacity of the rarely and frequently occurring mutants to replicate in a spreading infection assay in PBMC (Figure 10C). These results suggest that the rarity of apparently fit CA mutant viruses in natural HIV-1 subtype B populations is not due to differences in their capacity to replicate in primary cells. Rather, this finding suggests the presence of some unknown selective pressure that allows some intrinsically fit CA variants, but not others, to persist *in vivo*.

### Selective pressures that occur *in vivo* select sequence changes in the most genetically fragile domains of HIV-1 CA

**Figure 11:**
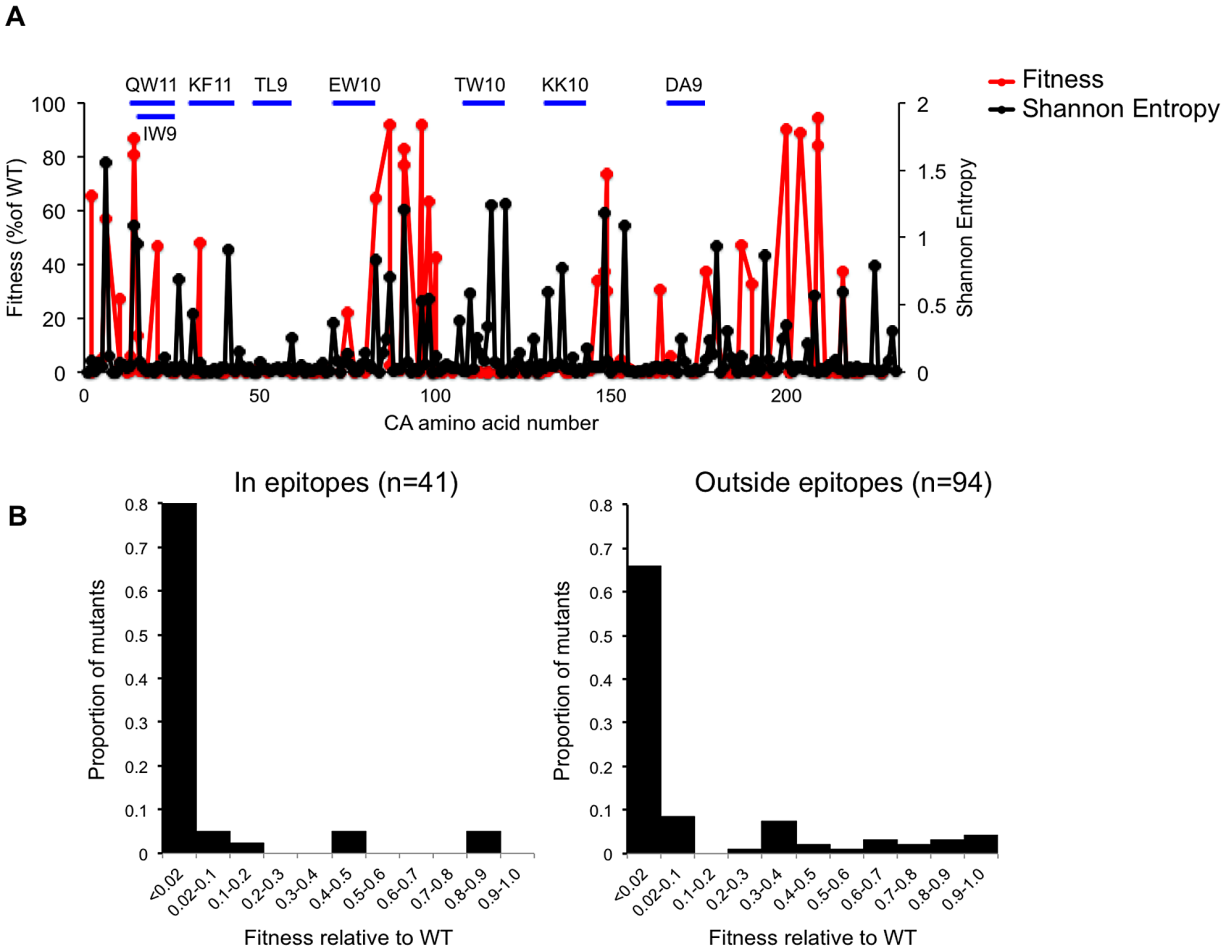
Correlation between fitness measurements and natural variation across the subtype B HIV-1 CA sequence. (A) Plot of fitness HIV-1 for library CA-mutants, overlayed with the Shannon entropy values for every residue in capsid, derived from 1000 subtype B isolates. The locations of the CA residues are arranged on the X-axis from left (N-terminal residue) to right (C-terminal residue), and their corresponding fitness (as a % of WT in a spreading replication assay) and Shannon entropy values are plotted on the Y-axes. The location of so called ‘protective’ CTL epitopes is indicated by horizontal blue lines. (B) Distribution of mutational fitness effects (DMFE) (at 37°C) for all single amino acid CA substitution mutants that occurred in or outside the protective epitopes indicated in (A).

To further examine the relationship between the impact of CA mutation on *in vitro* fitness and occurrence in natural HIV-1 subtype B populations, we next compared naturally occurring variability across the CA sequence (as measured by Shannon entropy value) with the propensity of CA domains to tolerate mutation. The expectation was that regions that are more robust (Figure 3A) should exhibit higher variability in natural populations. Like a previous analysis of the Shannon entropy of the N-terminal of subtype C HIV-1 [43], this analysis revealed regions of high entropy in CA (>0.4) while other regions were more conserved (Figure 11A). There was a degree of accord between the ability of CA domains to tolerate mutation and Shannon entropy values. For example, there were shared regions of comparatively high robustness and high entropy, such as the cyclophilin binding loop and regions encompassing residues ∼5 to ∼35, and ∼175 to ∼210. Additionally, there were regions where especially low robustness coincided with regions of low variability *in vivo*, such as in CA helices 2 and 3, and also in the MHR (Figure 11A). However, there were also significant discrepancies between the Shannon entropy and robustness profiles across CA. For example, in helices 5, 6, and 7 (from residues 101 to 145), Shannon entropy values were often quite high, but fitness measurements revealed this region to be highly genetically fragile. This finding strongly suggests that selective pressures in addition to fitness act on the HIV-1 CA sequence *in vivo*.

One such selective pressure is likely imposed by the adaptive immune system, particularly cytotoxic T-lymphocytes (CTLs) that can recognize peptides derived from HIV-1 CA. Indeed, several studies have demonstrated that mutations in CA can be driven by selective pressures imposed by CTLs [43]–[46]. Moreover, some of these studies have indicated that immune responses to particular epitopes in CA are protective, in that their appearance correlates with slower disease progression. For example, the oft studied protective HLA-B*57-restricted epitopes KF11 (residues 30–40) and TW10 (residues 108–117), and the B*27 restricted epitope KK10 (residues 131–140), all occur in regions of apparently extreme genetic fragility (Figure 11A, Table 2). In fact, a comparison of the DMFE values for the 41 library mutations that occurred in so-called protective epitopes [specifically QW11 (residues 13–23), IW9 (residues 15–23), KF11 (residues 30–40), TL9 (residues 48–56), EW10 (residues 71–80), TW10 (residues 108–117), DA9 (residues 166–174), KK10 (residues 131–140)] with 94 library mutations that occurred outside these epitopes, suggested that protective epitopes occur in regions of CA that are more genetically fragile than remaining CA sequence (Figure 11B). Indeed, the mean fitness value for mutations occurring within these ‘protective’ epitopes was 7.9% of WT while the mean fitness value for mutations occurring outside these epitopes was 15.1% of WT.

### Summary of the fates of CA mutations

**Figure 12:**
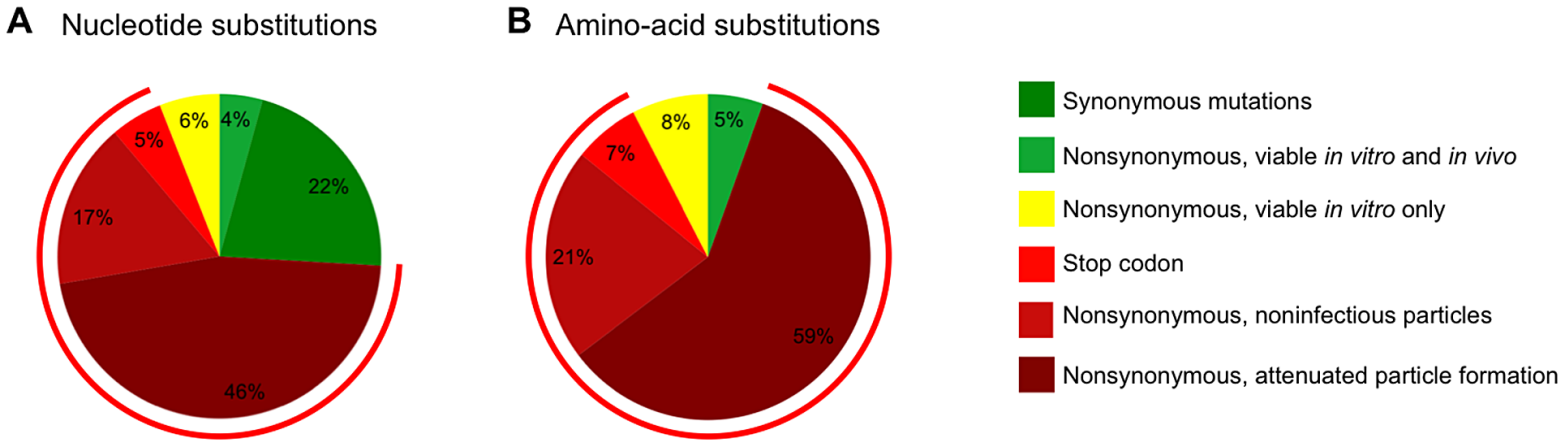
Summary of the effects of random mutations in HIV-1 CA. (A) Estimation of the frequency of various outcomes, based on *in vitro* and *in vivo* data, for random single nucleotide substitutions (A) and random single amino acid changes (B). The charts indicate changes that are synonymous (A only), nonsynonymous and nonsense (determined computationally using the CA sequence from pNHGcapNM). Mutants encoding stop codons in CA were assumed to be non-viable and synonymous changes were assumed to be viable. Because only mutants with >40% fitness *in vitro* are frequently observed in natural populations, this threshold was considered as sufficiently fit to thrive *in vivo* (the proportion of random mutants that exhibit less than this 40% fitness threshold was estimated using data from Tables 1 and 2, and is indicated by the red circumferential line). The proportion of mutants actually expected to thrive (nonsynonymous viable *in vitro* and *in vivo*) and expected not to thrive (nonsynonymous viable *in vitro* only) was estimated using the data from Figure 9. The fate of the remaining mutants was estimated using the data from Figure 6 (nonsynonymous, non-infectious particles or nonsynonymous, attenuated (>5-fold deficit) particle formation).

The scope of the mutagenesis carried out in this study, in which almost half of the amino acids in CA were individually mutated, coupled with an analysis of the frequency with which these mutations are present in natural populations, should allow generalized predictions of the fate of randomly introduced single nucleotide (Figure 12A) or single amino acid (Figure 12B) substitutions into CA. In the case of random nucleotide changes (Figure 12A), 22% should give synonymous changes that, in the great majority of cases, should not affect fitness (Figure 2). If there was no negative selection pressure *in vivo* other than fitness, then 10% of nucleotide substitutions are expected to yield a nonsynonymous change that is sufficiently replication competent (40% of *in vitro* WT fitness) to be potentially capable of flourishing in natural populations. However, if, as suggested by the data in Figure 9, it is true that only a fraction of mutations that yield variants with >40% of WT *in vitro* fitness are capable of persisting in vivo, then only 4% of all nucleotide substitutions are expected to yield non-synonymous changes that would flourish in a natural setting.

If only nonsynonymous nucleoside substitutions are considered (Figure 12B), then 87% are expected to be non-viable, with most mutations (59%) inducing a >5-fold attenuation of particle formation and a smaller fraction (21%) resulting in the generation of particles (at least 20% of the level of WT) that are non-infectious. Only 13% of nonsynonymous mutations are predicted to be sufficiently replication competent (at least 40% of WT fitness) to be potentially capable of flourishing *in vivo*, based on measurements of intrinsic fitness. Given the finding that only a fraction of intrinsically fit mutants actually persist *in vivo*, these data suggest that only 5% of all CA amino acid substitutions are actually expected to thrive in a natural setting.

## Discussion

The goal of this study was to generate a reasonably sized sample of random mutations in the CA protein that might arise naturally during HIV-1 replication, and examine their biological effects. In so doing, we could determine the genetic robustness of HIV-1 CA and correlate the effect of amino acid substitutions *in vitro* with their occurrence in natural viral populations. Moreover, such a large library constitutes a resource for investigating various functions and properties of the HIV-1 capsid. Our results uncover a rather extreme genetic fragility in the HIV-1 CA protein, with a large fraction (∼70%) of individual, random amino acid substitutions resulting in non-viable viruses (<2% of WT fitness). Tables 4 and 5 place these findings in context. Specifically, Table 4 summarizes all previous studies of randomly mutagenized HIV-1 proteins (and domains thereof), as well as all other randomly mutagenized viral proteins, and full viral genomes. Table 5 lists the genetic robustness of selected non-viral proteins (it is not inclusive of all random mutagenesis studies but we were unable to find any more fragile than those listed). Viruses (particularly ss(+) RNA viruses) not only have much higher mutation rates than cellular organisms, but also have comparatively low tolerance to mutation (Table 4,5) [61], [62]. Even when compared to viral genomes or proteins (e.g. enzymes, Table 5) that exhibit low robustness, HIV-1 CA is more genetically fragile than any other protein or virus for which this property has been measured.

**Table 4:** Comparison of the robustness/fragility of CA with other viral sequences*.

Protein/Domain	Fragility#	Author	Summary/Notes
**HIV**			
**HIV-1 capsid**	**70%**	**Rihn ** ***et al.***	**This study.**
HIV-1 protease flap region (aa 46–56)	61%	Shao *et al.* [87]	80 out of 131 mutants had negative activity, as shown by lack of Western blot p66/p51 bands
HIV-1 protease	54%	Parera *et al.* [88]	A total 86% of 107 single mutants were deleterious to catalytic efficiency, 54% lethal
HIV-1 protease	40%	Loeb *et al.* [89]	Out of 330 single missense mutants, around 40% were considered sensitive to mutation.
HIV-1 reverse transcriptase palm subdomain (aa 164–203)	28%	Chao *et al.* [90]	From 154 single mutants, 43 had RT levels that were ≤2% of WT
HIV-2 integrase	13%	van den Ent *et al.* [91]	5 out of 38 mutants display severely attenuated integration.
HIV-1 reverse transcriptase motif B	12%	Smith *et al.* [92]	Using infectivity results, 3 out of 25 mutants had ≤2% of WT levels
HIV-1 transcriptional promoter (LTR)	0%	van Opijnen *et al.* [93]	None of the 15 single mutants diminished infectivity as much as 2-fold in any cell line
HIV-1 reverse transcriptase (aa 67–78 of beta3-beta4 loop)	0%	Kim *et al.* [94]	Fragility measured by genetic selection in E. coli, in which no WT aa were essential
**Other viral proteins/domains**			
Human Papilloma Virus 16 E1	63%	Yasugi *et al.* [95]	5/8 single mutants were unable to replicate
Simian immunodeficiency virus mac239 envelope constant region 4	58%	Morrison *et al.* [96]	Only 11/26 single mutants were able to replicate appreciably
H3N2 influenza A hemaglutinin	56%	Nakajima *et al.* [97]	44% of 248 single aa changes did not abrogate hemadsorption activity
H2N2 influenza A neuraminidase	53%	Yano *et al.* [98]	47% of 152 single mutations in NA were tolerated, certain regions were more sensitive
Bacteriophage λ Cro	53%	Pakula *et al.* [99]	Approximately 37 out of 70 single residue mutants have ≤2% of WT CRM levels
Mason-Pfizer monkey virus matrix	50%	Rhee *et al.* [100]	4/8 single mutants had significant cell-free infectivity
Moloney murine leukemia virus capsid central domain	40%	Alin *et al.* [71]	Virus replication was considered blocked based on RT levels for 6 of 15 single mutants
Bacteriophage f1 gene V protein	35%	Terwilliger *et al.* [101] Masso *et al.* [102]	139 out of 371 single point mutants were inactive (unable to inhibit E. coli growth)
Varicella-Zoster virus thymidine kinase	35%	Suzutani *et al.* [103]	Out of 17 single aa mutants, 6 resulted in a loss of enzyme activity
Bunyavirus nucleocapsid (N)	4–26%	Eifan *et al.* [104]	5/115 single mutants had ≤2% WT mini replicon activity, 30/57 tested mutants had titers ≤2% of WT
Wheat streak mosaic virus HC-Pro	24%	Stenger *et al.* [105]	6 out of 25 nonsynonymous substitutions abolished systemic infectivity
Bacteriophage T4 lysozyme	16%	Rennell *et al.* [106]	From 2015 single aa substitutions, 328 were deleterious enough to prevent plaque formation
Mason-Pfizer monkey virus capsid MHR	15%	Strambio-de-Castillia *et al.* [107]	2/13 single mutants have RT levels at ≤2% of WT, all other mutants maintain at least 50%
Adenovirus VI	9%	Moyer *et al.* [108]	Only 1 out of 11 random single mutants led to a greater than 10-fold reduction in infectivity
Encephalomyocarditis virus internal ribosome entry site	0%	Van Der Velden *et al.* [109]	Zero out of 13 single random mutants had significantly impaired *in vitro* translation
**Whole Viral Genomes**			
Tobacco etch potyvirus (whole genome)	41%	Carrasco *et al.* [9]	Out of 66 single mutants, 41% were lethal and an additional 36% were deleterious
Vesicular stomatitis virus (whole genome)	40%	Sanjuán *et al.* [8]	Fitness analysis of 91 single mutants showed a further 29% of mutants were deleterious
Bacteriophage Qβ	29%	Domingo-Calap *et al.* [7]	29% of single mutations in 42 clones of Qβ were considered lethal
Bacteriophage f1 (whole genome)	24%	Peris *et al.* [110]	24% of a total 51 single missense mutants were lethal. Gene lethality varied 7 to 47%
Bacteriophage ΦX174	20%	Domingo-Calap *et al.* [7]	20% of single mutations in 45 clones of ΦX174 were considered lethal

* Table describes single residue mutagenesis studies that were (i) random (i.e. the cohort of mutants was not selected, and did not have amino acid bias) (ii) analysed proteins or domains greater than 10 aa in length, and (iii) had datasets that included at least 5 mutants.

# Fragility, when not stated clearly in the published work, was deduced using the % of mutants either indicated as ‘lethal’ or that had 2% or less fitness or function compared to WT, using the available analyses of fitness or function utilized in the study. In most cases, wording of summary/notes mimics the author's assessment as closely as possible.

**Table 5:** Robustness/fragility measurements in non-viral proteins*.

Other Proteins			
Protein/Domain	Fragility*^#^*	Author	Summary/Notes
E. coli lac repressor	<41%	Markiewicz *et al.* [111]	From 4000 single mutants, authors conclude 192 of 328 aa should be tolerant to substitution
3-methyladenine DNA glycosylase (AAG)	34%	Guo *et al.* [112]	The calculated probability of a single random amino acid change inactivating AAG is 34%
Campylobacter jejuni oligosaccaryltransferase PglB	33%	Ihssen *et al.* [113]	16 of the 24 single mutants maintain strong PglB expression by ELISA
Human interleukin-3	29%	Olins *et al.* [114]	From analysis of 770 mutants, 71% of residues were considered tolerant, as they had ≥5% of WT bioactivity
*Bacillus stearothermophilus* α-amylase	20%	Holm *et al.* [57]	12 out of 61 single random mutants had zero enzyme activity
TEM-1 beta-lactamase	16%	Huang *et al.* [115]	Authors conclude that 43 of the 263 residues cannot tolerate substitutions
T15 antibody heavy chain CDR2	7%	Chen *et al.* [116]	Just 1 out of 15 single CDR2 mutants lost binding function
Barnase, a bacterial ribonuclease	5%	Axe *et al.* [117]	5% (of over 600 mutants) were found to render barnase wholly inactive
Human interleukin-1α	2%	Kawashima *et al.* [118]	Only 1 out of 56 single mutants appeared to have significantly impaired activity

* Conditions for inclusion in the table were the same as those listed for Table 4.

The extreme mutational fragility of the HIV-1 CA may be related to the requirement that it maintain a structure that enables several different sets of interactions. Within the mature hexameric CA lattice, each CA monomer must interact, via distinct interfaces, with at least three other CA monomers, and adopt a rather tightly constrained position with respect to its internal structure as well as other CA monomers within a hexamer [26]. However, some CA monomers, that have precisely the same amino acid sequence, must be capable of adopting slightly different structures and positions relative to other CA monomers in order to form the pentameric declinations that enable closure of the CA lattice [16], [24]. Furthermore, a different set of CA:CA interactions are likely required during the formation of immature virions [15]. In addition to these constraints, the strength of the various CA:CA interactions in each of the different configurations probably needs to be finely tuned so that transitions from immature to mature virions during assembly, and appropriately timed disassembly, of the capsid can be accomplished in newly infected cells. Indeed, previous findings have suggested that appropriate capsid stability is required for HIV-1 infectiousness [63]. Additionally, CA interacts with a variety of host proteins, including cyclophilin A [23], ABCE1 [22] and possibly with nuclear pore complex proteins [37] and CPSF6 [34]–[36]. In summary, the number of CA interactions and functions, the requirement for CA to adopt several different structures, and transition between them in an ordered manner at multiple, critical stages of replication, likely places tight constraints on sequence and reduces the number of possible mutations that lack a major fitness cost.

Although lethal CA mutations could in principle affect any one of multiple steps in the HIV-1 life cycle, we found that most constitutively non-viable mutants exhibited attenuated particle formation. Moreover, all of the conditionally non-viable ts mutants exhibited attenuated particle formation at the non-permissive temperature. Interestingly, these deficits, at least for the conditionally non-viable mutants, could be rescued by inactivation of the viral protease. These results indicate that the critical role of CA in mediating efficient and accurate particle assembly, especially during Gag proteolysis, is the principal cause of its genetic fragility. How particle formation, budding and maturation are correctly orchestrated is not yet fully understood. Over a relatively short period of time (5–6 minutes) [64], thousands of Gag and Gag-Pol molecules coalesce and must be cleaved and rearranged through regimented and dynamically linked processes. Previous work has demonstrated that incorrect, premature activation of the HIV-1 protease can prevent particle formation [65]. Moreover, other perturbations of Gag (e.g. partial nucleocapsid deletion) can attenuate particle formation in a manner that is rescued by inactivation/inhibition of the viral protease [66], [67]. One idea that could explain these findings is that delayed particle formation may result in more extensive proteolytic cleavage of Gag early in the assembly process, thereby attenuating particle formation [66], [67]. The finding that many of the constitutively non-viable and all of the conditionally non-viable CA mutants showed evidence of perturbed Gag processing with additional ∼40 kDa protein species is consistent with the idea that CA mutations could similarly delay particle formation, resulting in mistiming of Gag proteolysis relative to budding and, therefore, protease dependent attenuation of particle formation. Within this framework, it is easier to understand how even subtle perturbation of Gag structure or multimerization caused by single amino acid substitutions could disrupt the fragile process of HIV-1 assembly, and thus how critical a role CA plays in orchestrating virion morphogenesis.

The high frequency with which mutations in the N-terminal domain of CA result in diminished particle production is intuitively at odds with earlier findings that the N-terminal domain of CA is dispensable for particle formation [68]. It has already been hypothesized that a misfolded N-terminus (caused by small changes in CA) could be more disruptive to assembly processes than deletion [68]. Even without overt misfolding, the presence of a CA NTD that does not pack correctly or efficiently into a hexagonal lattice is likely to be more disruptive than NTD removal to other interactions that are essential for particle formation (e.g. CA CTD-CTD interactions).

Previous large-scale mutagenesis studies of the retroviral CA proteins have employed insertional mutagenesis of MLV CA (using 12 amino acid peptide sequences) [69], [70] and alanine substitution of surface exposed residues in HIV-1 CA [56]. The fact that 12 amino acid insertions into MLV CA were uniformly lethal is consistent with the notion that retroviral CA proteins in general are sensitive to mutation, but insertions are obviously more likely to perturb CA structure than single amino acid substitutions. Indeed, a study of MLV CA mutants revealed that only 6 of 15 single amino acid substitutions (40%) in the central portion of CA were lethal [71]. Interestingly this study found that the most frequent phenotype associated with single amino acid substitutions in MLV CA was the generation of non-infectious particles rather than impaired particle formation, perhaps suggesting a difference in the properties of MLV and HIV-1 CA proteins. Previously, the most extensive mutagenesis study of HIV-1 CA [56] generated 48 alanine-scanning mutants that targeted surface exposed residues, of which 44% were non-viable. This study also found that single CA mutations in both the CTD and NTD can cause attenuated or aberrant particle formation, as well as the generation of morphologically normal virions that are non-infectious [56]. The difference in the fraction of mutations that were lethal in [56] versus our study is consistent with our finding that viable CA mutants tended to be confined to surface-exposed residues while mutation of the inner core of the CA structure was rarely compatible with viability. Most notably, we found that sequences encoding CA helices, particularly those in the NTD, were especially genetically fragile. These sequences form the fundamental structure of the CA hexamer, and in some cases (e.g. helices 1, 2 and 3), participate in CA:CA interactions within CA hexamers [26]. Mutations in CTD helices, and in the interhelical loops (e.g. the CypA binding loop) were less likely to cause lethal defects. This comparative tolerance of mutations in some portions of CA appears to correlate, at least partially, with conformational variation in crystal structures of CA hexamers [26]. In summary, CA NTD helices appear to be highly structurally constrained and particularly sensitive to mutations that might perturb the shape or stability of the CA NTD monomer or the CA hexamer.

By correlating our fitness measurements with an analysis of the frequency with which mutations occur in natural populations, we hoped to make observations that neither approach alone could yield, and make predictions about the outcomes of hypothetical mutations. For example, all of the mutations that were present in our library and occurred at a frequency of greater than 3% in natural populations (n = 9) were quite fit *in vitro* (>40% of WT fitness). Conversely, all mutants that exhibited a fitness of <20% of WT (n = 106) occurred at a frequency of <1% in natural populations. This finding indicates that there is some correlation between fitness and occurrence in natural populations, and suggests that below an ∼40% fitness threshold, mutations rarely confer a selective advantage within a human host. However, the correlation between mutant fitness and natural occurrence was clearly imperfect, indicating the impact of selective pressures other than fitness on naturally occurring sequences. Specifically, there was a lack of concordance in fitness and Shannon entropy profiles in some portions of the CA sequence, and it was striking that more than half of the mutations that had little or no impact on fitness, did not occur in natural populations (‘fit but rare’ mutants). This finding suggests the absence of a selective pressure driving the emergence of ‘fit but rare’ mutants, or perhaps the presence of a selective pressure against their emergence. Importantly, we were unable to demonstrate any defect in the ability of fit but rare CA mutants to infect primary cells, including primary T-cells and macrophages. One possible explanation for our findings is that fit but rare mutants are purged from natural populations because they confer sensitivity to adaptive or innate immune responses. Overall, our findings suggest that only ∼5% of all possible amino acid substitutions in CA result in viruses that can flourish *in vivo*.

An obvious candidate for selective pressure that induces sequence variation in regions of high genetic fragility, and could potentially suppress ‘fit but rare’ mutants is that imposed by CTLs. Indeed, a number of examples of CTL escape mutations in protective epitopes are characterized by (i) their predictability (i.e. recurrence of the same mutation in different infected individuals) [72], (ii) sometimes by their impact on viral fitness [45], [73], and (iii) co-occurrence with compensating mutations, suggesting an impact of epistatic interactions that alleviate compromised fitness induced by primary escape mutations [43], [44]. All of these findings are consistent with, and indeed explained by, the finding that CA is highly genetically fragile, and suggest that the number of possible mutations that enable escape from CTL responses, with retention of fitness, is small. However, in considering these arguments, it is important to recognize that even though our sample of CA mutants should be representative of random variation, it is only a sample of the possible mutations that can arise in CA in natural populations. Analyses in cell culture suggest that a single cycle of HIV-1 replication (incorporating transcription and reverse transcription steps) generates 1.4 to 3.4×10^−5^ mutations per nucleotide [74], [75]. For the 231 aa CA protein, this would result in approximately 0.0097–0.023 errors per replication cycle, or an amino acid substitution about once or twice per one hundred replication events. Importantly, the volume and rate of HIV-1 replication *in vivo* [76] means that every amino acid substitution that is accessible by a single nucleotide substitution is generated in nearly every infected individual, many times, every day. Thus, selective pressures imposed *in vivo* can act on an enormous pool of possible variants. The discrepancies between the fitness and Shannon entropy profiles in Figure 11 suggest that even in CA domains in which we found few or no fit mutants in our sample of 135 mutants, there do exist rare variants that can thrive *in vivo*, even if sometimes they exhibit compromised fitness or require the co-occurrence of compensating mutations.

Previous work has indicated that the CTL responses to Gag, rather than other HIV-1 proteins, correlate best with reduced viral burden during HIV-1 infection [49]. Notably, so-called ‘protective’ CTL epitopes [44] tended to occur in regions of CA that were even more intolerant of mutation than CA as a whole. Additionally, Gag is among the most abundant viral proteins expressed in HIV-1 infected cells. Its abundance, and the comparative lack of plasticity in CA, likely both contribute to the relatively favorable clinical outcome in patients that mount strong immune responses to the Gag protein [49]. Nevertheless, it appears that the ability of viral populations to explore essentially all possible amino acid substitutions, partly overcomes the genetic fragility of CA and enables at least some degree of escape from immune responses, even those that target the most vulnerable areas of CA.

Understanding the fitness effects of mutations in CA may facilitate drug or vaccine design. For example, one recent study done in the context of a possible vaccination strategy highlighted the observation that mutations in the most conserved residues in CA are not necessarily associated with the highest fitness costs [77], as might otherwise be assumed. Thus, a better understanding of the constraints under which CA evolves may prove beneficial. Additionally, while CA has not yet been exploited as a target for drugs in the clinic, recent findings have indicated that it is possible to do so, at least in principle [78]–[82]. The genetic fragility exhibited by CA should reduce, but clearly not eliminate, the opportunities for drug resistance to occur, and it would be especially desirable to target CA domains that exhibit the greatest fragility. In spite of this, resistance to one recently identified CA-targeted inhibitor arises *in vitro* through mutations in regions of CA that exhibit high fragility [79]. Again, it appears that the sheer number of variants that can be ‘tested’ in natural viral populations can, at least sometimes, overcome the lack of robustness of HIV-1 CA. Nevertheless, the high genetic fragility exhibited by CA should facilitate attacks on HIV-1 through vaccination or therapy.

## Materials and Methods

### Plasmids and construction

A mutagenized CA library was generated using the Genemorph II random mutagenesis kit (Agilent) using the following oligos 5′-GTA AGA AAA AGG CAC AGC AAG CGG CCG CTG -3′ and 5′- CTT GGC TCA TTG CTT CAG CCA AAA CGC GTG-3′. The PCR product was cloned using a TOPO TA Cloning Kit and plasmid DNA was extracted from approximately ∼1×10^4^ pooled, insert-positive colonies. After sequencing the amplicon from 20 clones to obtain a preliminary estimate of the mutagenesis frequency, this pooled plasmid DNA was digested using *Not*I and *Mlu*I and the CA-library insert subcloned into the pNHG (JQ585717) derivative pNHGCapNM (JQ686832, Figure 1A). The *Not*I and *Mlu*I digested proviral plasmid was prepared in advance and generated >200-fold fewer colonies when ligated without insert than when ligated with insert. Furthermore, representative restriction digests indicated that aberrant restriction patterns, perhaps due to recombination, occurred at a frequency of only 1–2% of clones. Proviral plasmid DNA was extracted from individual cultures derived from 1056 colonies and subjected to sequencing and further analysis. Proviral plasmid DNA from the single mutants listed in tables 1 and 2 was freshly isolated, re-sequenced and analyzed by *Not*I and *Mlu*I restriction digest.

The pNL4-3 Pr- plasmid is derived from pNHGCapNM and pNL4-3 (NIH AIDS Research and Reference Reagent Program, Catalog No. 114), and has *Not*I and *Mlu*I sites flanking CA as well as a point mutation in the protease active site, as has been previously described [83]. CA sequences harboring the temperature sensitive mutations were transferred from the pNHGcapNM vector to pNL4-3 Pr- by digestion with *Not*I and *Mlu*I.

### Cell lines and transfection

The adherent human cell lines, 293T and TZM-bl, were maintained in DMEM supplemented with 10% fetal calf serum (FCS) and gentamicin. Suspension MT-4 cells were maintained in RPMI with 10% FCS and gentamicin.

For transfection experiments, 293T cells were plated at 2.5×10^4^ cells/well in 96 well plates or 1.5×10^5^ cells/well in 24 well plates. To measure the effect of CA mutations, and their temperature sensitivity, transfections were done the following day using polyethylenimine (Polysciences), and either 100 ng of the WT or mutant NHGCapNM plasmids described above (for 96 well plate experiments) or 500 ng (for 24 well plate experiments), or 500 ng of the pNL4-3 Pr- mutants (all in 24 well plate experiments). Plates for all transfection experiments were placed at 35, 37 or 39.5°C, as indicated, immediately upon addition of transfection mixture.

### Primary cells

PBMCs, CD4+ T cells, and macrophages were isolated from buffy coats (from anonymous healthy blood donors and were purchased from the New York Blood Center) using a Ficoll gradient. Primary CD4+ T cells were extracted using a RosetteSep Human CD4+ T-cell enrichment cocktail. Macrophages were isolated by adhesion to plastic and treatment with 100 ng/ml of granulocyte/macrophage-colony stimulating factor (GM-CSF) for 96 hours prior to infection. All primary cells were maintained in RPMI supplemented with 10% FCS, penicillin/streptomycin, and L-glutamine. Activation of PBMCs and CD4+ T cells was achieved by addition of phytohemagglutinin (PHA) at 5 µg/ml for 72 hours prior to infection, with addition of 25 U/ml of interleukin-2 at the time of infection.

### Viral replication and infectivity assays

293T cells were transfected with the proviral plasmids and given fresh medium 16 hrs later. At ∼40 h post-transfection, cell supernatants were collected and filtered (0.22 µm). Single-cycle infectivity was measured using MT-4 cells that were seeded in 96 well plates at 3×10^4^/well and inoculated with a volume of filtered supernatant that was equivalent to an MOI of ∼1 for the WT viral clone. Dextran sulfate at 100 µM was added 16 hrs later to limit replication to a single cycle, and cells were fixed in 4% PFA 48 hrs after infection. Alternatively, harvested supernatants were subjected to a low-speed spin and a freeze-thaw cycle before addition to MT-4 or TZM-bl cells in 96 well plates, in the presence or absence of aphidicolin (2 µg/ml) or at 35, 37, or 39.5°C, as indicated in figure legends. For spreading replication assays, MT-4 cells were inoculated with a volume of filtered supernatant that was equivalent to an MOI of ∼0.01 for the WT viral clone and fixed in 4% PFA at 72–80 hrs post-infection. FACS analysis for all infectivity and replication assays was carried out using a Guava EasyCyte instrument.

The following mutations, some of which were part of either double or triple mutants, occurred in mutants that exhibited temperature sensitivity in spreading replication assay (Figure S1): S4C, L189M, I91F; Q6P, A78V; I15M, A78T; S16T, T48A; S33C, A92V; I91N, D163E; I91V, I124T; M96T; R100S, Q112L; R132G; R167Q; M214L; K227I. CA mutants encoding each individual substitution were generated, as necessary, prior to selection of the 11 ts mutants for analysis in Figures 4 and 5.

Infections of primary cells required different conditions. For single-cycle infections of PBMCs and CD4+ T cells, 0.1–1×10^6^ cells were inoculated using virus generated from 293T cells, as described above, for the 11 rarely occurring mutants described in Figure 9C, the WT virus, and 4 randomly selected frequently occurring mutants from Figure 9B (H87Q, I91V, E98D, T200S) at an MOI of ∼5, and were fixed in 4% PFA 36 hours later. For infection of macrophages, VSV-pseudotyped virus was generated in 293T cells for the same 16 mutants plus WT virus. Macrophages were infected at an MOI of ∼4 and fixed approximately 72 hours later. For spreading infection assays in PBMC, 1×10^6^ cells were infected at an MOI of 0.1, and cells fixed at the time points indicated in Figure 10C. All MOIs used for primary cells were reference values derived from titrations on MT-4 cells.

### Western blotting

Cell lysates and virions (pelleted by centrifugation through 20% sucrose) were resuspended in SDS sample buffer and separated by electrophoresis on NuPage 4–12% Bis-Tris Gels (Novex). Proteins were subsequently blotted onto nitrocellulose membranes, probed with a primary anti-HIV p24 capsid antibody (183-H12-5C) or anti-HIV p17 antibody (VU47 Rabbit anti-p17 [84]), and then probed with a goat anti-mouse/anti-rabbit IRDye® 800CW secondary antibody (LI-COR Biosciences). A LI-COR Odyssey scanner was used to detect and quantify fluorescent signals. A minimum of 3 separate Western blots was produced for each temperature sensitive mutant, including those in the protease negative background, and representative blots are shown.

### Structure analysis

HIV-1 capsid hexamer structural analysis was done using MacPyMOL, with PDB reference 3GV2 [26]. Solvent accessibility surface area of residues was determined using UCSF Chimera.

### Thin-section electron microscopy

To prepare samples for thin-section electron microscopy, 293T cells were seeded in 6-well plates at 0.8×10^6^ cells per well in duplicate. Transfections were done the following day with the addition of polyethylenimine to 2 µg of the WT or mutant NHGcapNM plasmids (S33C, T48A, M96T, Q112L, R132G, L189M, G60W, K25I, L52F or N195S) plus 0.5 µg of modified human tetherin (delGI, T451 that is resistant to antagonism by HIV-1 Vpu [58]) to enhance visualization of virions at the plasma membrane. Immediately after addition of transfection mixture, plates were placed at either 37 or 39.5°C, as indicated. After 48 hrs, supernatant was removed and cells were fixed in a solution of 2% paraformaldehyde (PFA), 2.5% glutaraldehyde. One set of cells was then analyzed by FACS to compare transfection efficiencies (which ranged from 23% for WT to 32% for L189M). The other set were fixed with 2.5% glutaraldehyde and 1% osmium tetroxide, and stained with 2% aqueous uranyl acetate. Fixed and stained cells were harvested into PBS and pelleted through 1% SeaPlaque agarose (Flowgen) at 45°C. The agar was set at 4°C and the cell pellets were cut into ∼2 mm cubes, which were dehydrated through a graded alcohol series and infiltrated with TAAB 812 embedding resin. After polymerisation, thin sections (120 nm) were cut and examined in a JEOL 1200 EX II electron microscope. Numbers of virus particles associated with 150 randomly selected cells were counted for each sample.

### Cryo-electron microscopy

To isolate virions for cryo-electron microscopy, 10 cm plates of approximately 4×10^6^ 293T cells were transfected with 7 µg of the indicated plasmids (NHGcapNM, R18G, K30N, G60W or M215V) using polyethylenimine. After 48 hours, supernatant was collected and filtered and virions were pelleted by centrifugation at 14000 rpm through 20% sucrose. Virions were fixed in 10 µl of 2% PFA, 2.5% glutaraldehyde solution.

Fixed aliquots of 3 ul of each sample were loaded onto freshly glow-discharged c-flat holey carbon grids (CF-22-4C, Protochips, Inc.) held at 4°C and 100% humidity in a Vitrobot vitrification robot (FEI). Grids were blotted for 4 s prior to being frozen by plunging into a bath of liquid nitrogen-cooled liquid ethane. Vitrified specimens were imaged at low temperature in a JEOL 2200 FS cryo-microscope equipped with Gatan 626 cryo-stages. Low dose (10 e/Å2), energy-filtered images (slit width, 20 eV) were recorded on a Gatan ultrascan 16-megapixel charge-coupled-device camera at a magnification of 50,000×.

### Analysis of natural CA variants

A set of 1,000 HIV-1 subtype B sequences isolates was obtained from the Los Alamos HIV sequence database (www.hiv.lanl.gov/). All sequences were sampled from distinct infections between from 1980 and 2009. To minimize risks of sampling biases, multiple sequences from known transmission clusters were excluded. Sequences with frameshift mutations or stop codons that were likely to represent nonfunctional viruses or poor quality sequencing were excluded. Sequences were aligned using MUSCLE [85], and PERL scripts were used to examine genetic variation in the resulting sequence alignment. An information-theoretic measure of diversity (Shannon's entropy) [86] was applied to quantify the amount of amino acid variation at each position in capsid.

## Supporting Information

Figure S1 Temperature sensitive mutants in entire CA mutant library. The Y-axis indicates the percentage of infected (GFP+) MT-4 cells from mutant viruses following a spreading replication assay done at 35°C, while the x-axis shows the percentage of infected cells following a spreading replication assay done at 39.5°C. Mutants that generated less than 0.1% infected cells are not shown.
(TIF)
