# Supplementary figures and images for: Extreme Genetic Fragility of the HIV-1 Capsid
Source: PLoS Pathog. 2013 Jun 20;9(6):e1003461. doi: 10.1371/journal.ppat.1003461 (PMC3688543; doi:10.1371/journal.ppat.1003461)

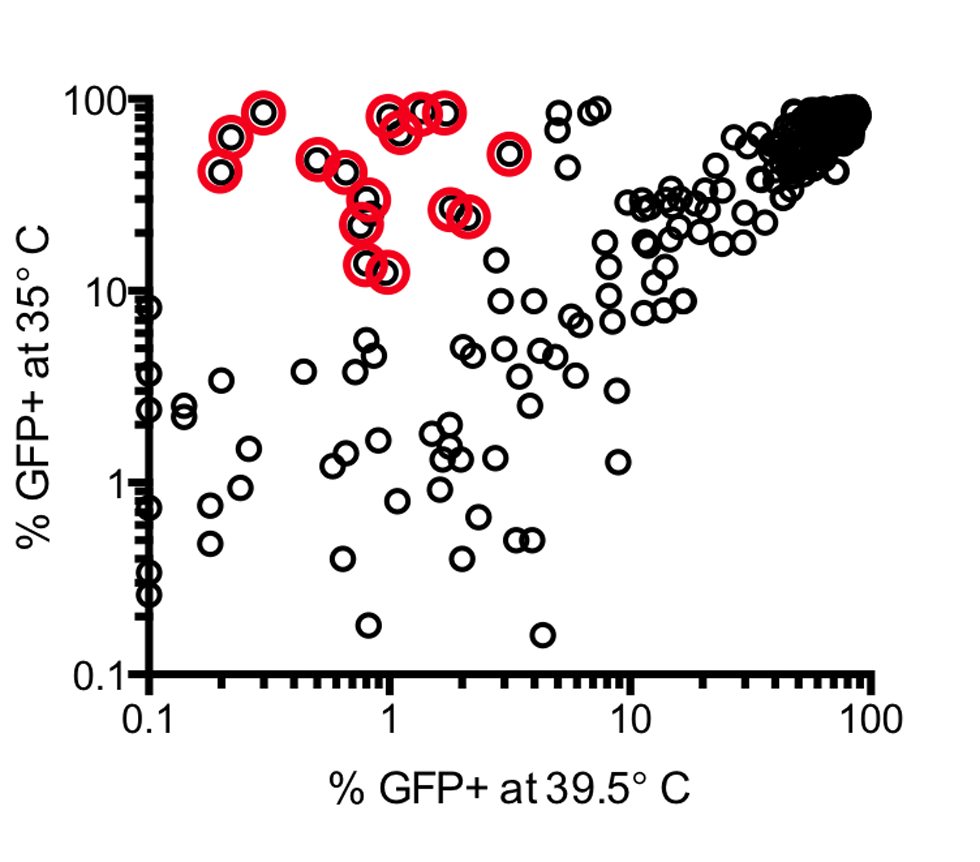

Supplement: Figure S1 — Temperature sensitive mutants in entire CA mutant library. The Y-axis indicates the percentage of infected (GFP+) MT-4 cells from mutant viruses following a spreading replication assay done at 35°C, while the x-axis shows the percentage of infected cells following a spreading replication assay done at 39.5°C. Mutants that generated less than 0.1% infected cells are not shown. (TIF) [file ppat.1003461.s001.tif]
